# Hypertonic saline (HS) for acute bronchiolitis: Systematic review and meta-analysis

**DOI:** 10.1186/s12890-015-0140-x

**Published:** 2015-11-23

**Authors:** Chin Maguire, Hannah Cantrill, Daniel Hind, Mike Bradburn, Mark L. Everard

**Affiliations:** Clinical Trials Research Unit, University of Sheffield, Sheffield, UK; School of Paediatrics and Child Health (SPACH), The University of Western Australia, Perth, Australia

**Keywords:** Bronchiolitis, Bronchodilator agents, Nebulizers and vaporizers, Randomized controlled trials as topic, Hypertonic saline, Systematic review, Meta-analysis

## Abstract

**Background:**

Acute bronchiolitis is the commonest cause of hospitalisation in infancy. Currently management consists of supportive care and oxygen. A Cochrane review concluded that, “nebulised 3 % saline may significantly reduce the length of hospital stay”. We conducted a systematic review of controlled trials of nebulised hypertonic saline (HS) for infants hospitalised with primary acute bronchiolitis.

**Methods:**

Searches to January 2015 involved: Cochrane Central Register of Controlled Trials; Ovid MEDLINE; Embase; Google Scholar; Web of Science; and, a variety of trials registers. We hand searched Chest, Paediatrics and Journal of Paediatrics on 14 January 2015. Reference lists of eligible trial publications were checked. Randomised or quasi-randomised trials which compared HS versus either normal saline (+/− adjunct treatment) or no treatment were included. Eligible studies involved children less than 2 years old hospitalised due to the first episode of acute bronchiolitis. Two reviewers extracted data to calculate mean differences (MD) and 95 % Confidence Intervals (CIs) for length of hospital stay (LoS—primary outcome), Clinical Severity Score (CSS) and Serious Adverse Events (SAEs). Meta-analysis was undertaken using a fixed effect model, supplemented with additional sensitivity analyses. We investigated statistical heterogeneity using I^2^. Risk of bias, within and between studies, was assessed using the Cochrane tool, an outcome reporting bias checklist and a funnel plot.

**Results:**

Fifteen trials were included in the systematic review (*n* = 1922), HS reduced mean LoS by 0.36, (95 % CI 0.50 to 0.22) days, but with considerable heterogeneity (I^2^ = 78 %) and sensitivity to alternative analysis methods. A reduction in CSS was observed where assessed [*n* = 516; MD −1.36, CI −1.52, −1.20]. One trial reported one possible intervention related SAE, no other studies described intervention related SAEs.

**Conclusions:**

There is disparity between the overall combined effect on LoS as compared with the negative results from the largest and most precise trials. Together with high levels of heterogeneity, this means that neither individual trials nor pooled estimates provide a firm evidence-base for routine use of HS in inpatient acute bronchiolitis.

**Electronic supplementary material:**

The online version of this article (doi:10.1186/s12890-015-0140-x) contains supplementary material, which is available to authorized users.

## Background

Acute bronchiolitis is the most common cause for hospitalisation in infancy and childhood, with 1–3 % of all infants admitted to hospital during their first winter [1–9]. Obstruction of the airways results in severe breathing difficulties caused by common respiratory viruses infecting the lungs [1–8, 10–13]. The peak age of incidence is between 1 and 6 months for babies admitted with this condition [9]. Current management involves supportive care, minimal handling, supplemental oxygen and fluids [4, 14–16]. The median duration of admission is 3 days, considerably higher than for other acute paediatric admissions (median 1 day). The course of the illness or length of hospital stay has not been impacted by treatments including oral and inhaled steroids, antiviral agents and a variety of bronchodilators.

A number of small studies have suggested that nebulised hypertonic saline may influence the course of the illness resulting in a reduction in the duration of hospitalisation for infants admitted with “acute bronchiolitis” [17–20]. A Cochrane review, which last updated its searches in May 2013, included 11 trials involving 1090 infants with mild to moderate acute viral bronchiolitis (500 inpatients, six trials; 65 outpatients, one trial; and 525 emergency department patients, four trials) [21]. The review concluded that “current evidence suggests nebulized 3 % saline may significantly reduce the length of hospital stay and improve the clinical severity score in infants with acute viral bronchiolitis.” Our team felt that this conclusion is difficult to justify on two counts. Firstly, the findings are hampered by high levels of heterogeneity, and we believe these warrant further exploration. Secondly, a number of relevant published trials, published at the time appear to have been overlooked; this is still the case as of the 2013 update. Finally, since 2013, a number of large studies addressing this topic have been published.

As a result, we made a registration on the PROSPERO database and undertook a separate systematic review on the effect of hypertonic saline on the length of stay of infants admitted to hospital for acute bronchiolitis. The PROSPERO protocol is available (Additional file 1). This review does not investigate the use of hypertonic saline in infants with bronchiolitis in the emergency department.

## Methods

This study is reported in accordance with the Preferred Reporting Items for Systematic Reviews and Meta-Analyses (PRISMA) guidance [22]. A checklist is available (Additional file 2). Since this study was a literature review of previously reported studies, ethical approval or additional consent from participants was not required.

### Protocol and registration

The protocol was registered with the PROSPERO database (CRD42014007569) [23] on 03 March 14 and the registration was updated on 18 December 2014.

### Eligibility criteria

We included published and unpublished randomised controlled trials involving children up to the age of 2 years, hospitalised as the result of a first episode of acute bronchiolitis. Trials were included if hypertonic saline versus either normal saline (+/− adjunct treatment) or no treatment were the interventions. Studies were grouped in pre-specified subgroups as follows: (1) Nebulised hypertonic saline alone vs normal saline; (2) Nebulised hypertonic saline plus a bronchodilator (e.g. salbutamol) vs. normal saline; (3) Nebulised hypertonic saline plus a bronchodilator vs. normal saline plus same bronchodilator; (4) Nebulised hypertonic saline alone or plus a bronchodilator vs. no intervention. We applied no restrictions based on the concentration, dose or administration of the intervention or control. We excluded studies not published in English.

### Literature search and information sources

We searched Cochrane Central Register of Controlled Trials (CENTRAL), MEDLINE (via Ovid), EMBASE from inception to January 2015, Web of Science from 2010 to January 2015 and Google Scholar. We used 2010 as a cut off, the date on which Zhang and colleagues ran their searches for the 2011 update [24]. We used the terms “bronchiolitis” and “hypertonic saline” to identify ongoing unpublished data in the following registries: Clinicaltrials.gov; UK Clinical Trials Gateway (UKCTG); CRD databases (DARE NHS EED, HTA); controlled-trials.com; centrewatch.com and National Research Register (NNR). On 14th January 2015 we also hand-searched *Chest*, *Paediatrics* and *Journal of Paediatrics* using the terms “hypertonic saline” and “bronchiolitis”. One reviewer (HC) checked the reference lists of all eligible trial publications for other relevant trials. The final search strategy is outlined in Additional file 3.

### Study selection

Two reviewers (CM and HC) independently assessed eligibility, differences were resolved through discussion with third reviewers (DH and ME). Where the titles or abstracts suggested eligibility, we retrieved and screened the full paper. Unsuccessful attempts were made to contact trial investigators of three unpublished studies [25–27] to request additional unreported data.

### Data extraction

CM and HC used a standardised data collection tool to include study characteristics, population characteristics and risk of bias [28]. Population and study data comprised of country where the trial was conducted, age, disease severity, intervention and comparator details (concentration, dose, delivery mechanism), and details of any adjunct treatments given (β_2_ agonist or epinephrine). We extracted baseline and follow-up outcome data for Length of hospital stay (LoS), final Clinical Severity Scores (CSS) using the scoring system described by Wang and colleagues [29], readmission rate and adverse events. Adverse event data was collected however reported but of particular interest were tachycardia, hypertension, pallor, tremor, nausea, vomiting and acute urinary retention. DH helped resolve any discrepancies with any of the data items.

### Risk of bias

We separately assessed the potential for systematic error within individual studies using the Cochrane risk of bias tool [30] and the following dimensions of methodological quality: (1) generation of allocation sequence; (2) allocation concealment; (3) blinding (participant and researchers); (4) blinding of outcome assessors; (5) completeness of outcome data; (6) selective outcome reporting. Studies were grade as being at, “low”, “high” or “unclear” risk of bias. Any discrepancies were discussed between the data extractors until both reached a unanimous decision. Where an unclear grading was given we contacted trial authors to obtain further information and searched for the study protocol to identify sources of reporting bias. We used standard methods (based on the Cochrane Handbook) to assess funnel plot symmetry as we had greater than 10 trials in the meta-analysis [30]. We employed methods suggested by Dwan and colleagues [31] to assess the risk of outcome reporting bias for each of the outcomes using the Outcome Reporting Bias (ORB) classification whereby trials are scored as “high risk”, “low risk” or “partial risk”. The full classification table is available in Additional file 4.

### Synthesis

The primary outcome was LoS. Secondary outcomes were adverse events, final CSS scores and rate of readmission. Data on LoS and final mean CSS was used to calculate mean differences (MD) with 95 % Confidence Intervals (CIs) for each outcome. Where trials included arms using different concentrations of hypertonic saline in addition to a control arm we treated them as two separate trials, dividing the control arm numbers by two in order not to double-count control participants. Meta-analyses were undertaken in RevMan Version 5.2 and Stata version12 using both fixed (Inverse-Variance) and random effect (Der Simonian & Laird) models [32]; additional sensitivity analyses used the metasens package as implemented within the R software version 3.1.3[33]. The four pre-specified intervention subgroups were separated in the main forest plot to give subgroup estimates of treatment effects. Statistical heterogeneity—a measure of within-trial variation—was investigated using the I^2^ statistic [30]: 0–40 % indicating unimportant levels; 30–60 % showing moderate heterogeneity; 50–100 % as demonstrable heterogeneity within trials. Sensitivity analyses were undertaken as proposed by Deschartres et al. [34] along with meta-regression to assess whether heterogeneity could be attributed to measurable sources. An assessment for the potential of publication bias was made via assessment of the funnel plot generated for all trials included in the meta-analysis. We produced a narrative description of adverse events and readmission rates.

## Results

### Searches and selection

**Fig. 1:**
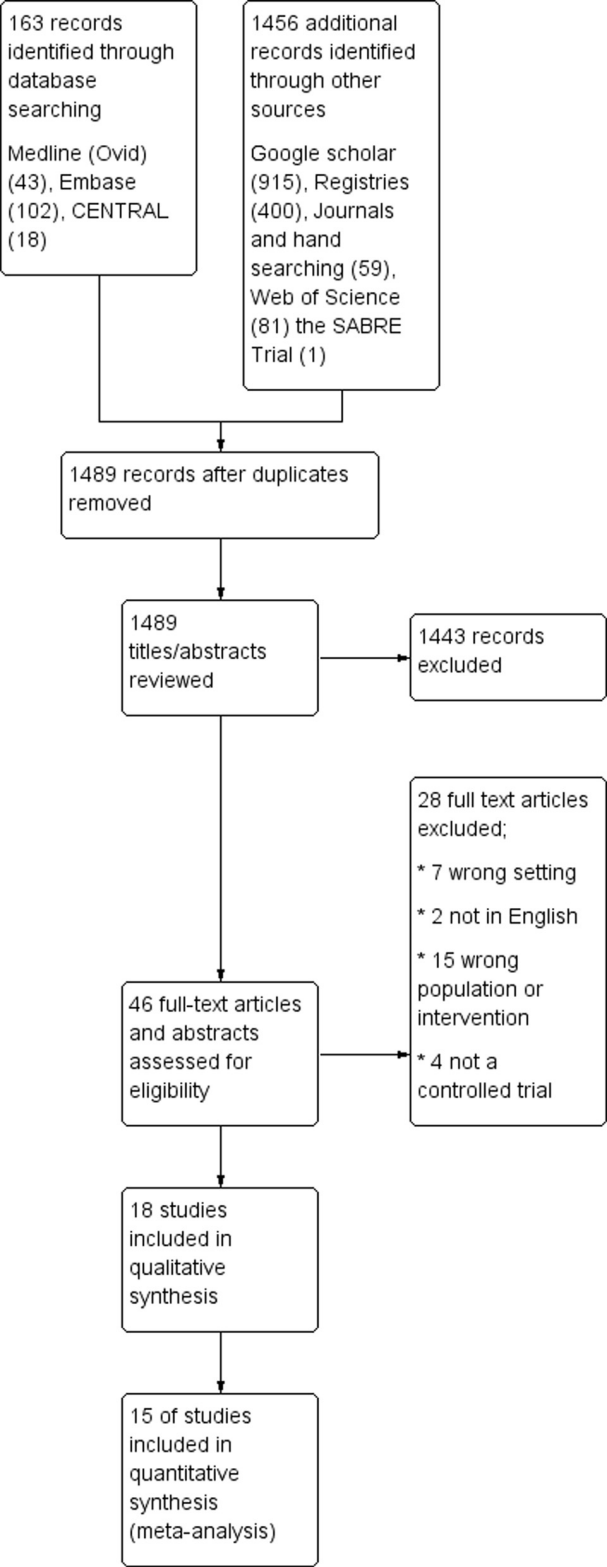
Study flow diagram

After removal of duplicates, the searches yielded 1489 citations, from which 1443 were excluded at the title/abstract stage (Fig. 1). We retrieved 46 full papers for further review of which 28 were excluded because they were not a controlled trial (*n* = 4) [35–38], were in the wrong setting (*n* = 7) [39–45] or wrong population or intervention (*n* = 15) [17, 46–59], or were not available in English (*n* = 2) [60, 61]. All included studies were parallel group, participant-randomised trials. 3 were multi-centre; 9 were single centre and the number of centres was unknown for 6. We identified 18 trials (2225 participants, excluding one trial as the number of participants is unknown [26]) eligible for inclusion at the full paper review stage (Fig. 1). Details of all excluded trials at the full stage can be found in Additional file 5.

### Study characteristics

The remaining 18 trials (total number of children included in the analysis) were conducted in: Italy (*n* = 106) [62]; United Arab Emirates and Canada (*n* = 91) [20]; China (*n* = 205) [63, 64]; Israel (*n* = 93) [18, 19]; Argentina (*n* = 82) [25]; India (*n* = 388) [65–67]; Qatar (*n* = 171) [68]; Georgia (*n* = 42) [69]; The Netherlands (*n* = 247) [70]; USA (*n* = 190) [71]; Turkey (*n* = 69) [27]; Mexico (*n* = unknown) [26]; Nepal (*n* = 59) [72] and UK (*n* = 290) [73] (Additional file 6). The sample size ranged from 40 [67] to 317 [73] participants. Eligibility criteria varied significantly in terms of patient characteristics and disease severity (Additional file 7). The upper age limit for subjects ranged from 12 to 24 months. Amongst the range of clinical characteristics required for inclusion were ‘bronchiolitis with temperature >38 °C’ , ‘first episode of wheezing with evidence of viral infection’ , ‘wheezing’ , ‘wheeze and/or crackles’ , ‘crackles’ , and ‘first episode of bronchiolitis’. In terms of severity, oxygen saturation in air was specified in 4 studies and ranged from <97 to <92 %. Numbers of participants allocated to each group was clearly defined in all but three trials [26, 27, 69] whose data was not usable in the meta-analysis as attempts to contact the author were fruitless.

### Population characteristics

The average age ranged from 2.6 months [19] to 8.6 months (median = 4.5 months). Five studies including children under 12 months age only [18, 19, 65, 70, 72]. The heterogeneity of study design and patient populations was emphasised by the indicators of disease severity at entry to the study. Baseline oxygen saturations were reported in nine studies [18–20, 62, 65, 66, 68, 70, 72]; these ranged from very mild (SaO_2_ of 90.5 % [64]) to significant respiratory compromise (SaO_2_ of 97.4 % [68]). Clinical scoring systems were used to classify disease severity in 15 of 18 studies [18–20, 26, 27, 62–68, 70–72]. Based on the scores at entry, the disease severity varied from mild/moderate [*n* = 5] [27, 63, 67, 69, 70], moderate [*n* = 5] [18–20, 25, 65],moderate to severe [*n* = 4] [26, 64, 66, 68] and severe [*n* = 2] [62, 73] confirming the heterogeneity of disease severity across the studies. A severity classification was not provided for Ojha et al. and Silver et al. [71, 72], but based on their characteristics were subjectively classified “severe” and “mild/moderate” respectively. Disease severity was assessed in fifteen of the 18 trials using a variety of scoring systems to characterise participants as “mild”, “moderate” or “severe” or a combination of each; Wang et al. CSS scoring system [29] (*n* = 8) [18, 19, 62–65, 67, 70], Respiratory Distress Assessment Instrument (RDAI) score (*n* = 3) [20, 66, 71], Bronchiolitis Severity Score (*n* = 1) [68], Bronchiolitis Clinical Score (*n* = 1) [69], Respiratory assessment score (*n* = 1) [27], Respiratory Distress Scale Sant Joan de Deu Hospital (*n* = 1) [26] and Clinical scoring of respiratory distress (*n* = 1) [72] (Table 1).

**Table 1:** Population characteristics

Study	Age—mean (SD)	Gender	Disease severity	Length of hospital stay mean (SD) (days)	Final CSS score
Al-Ansari 2010 et al. [68]	Intervention (3 % HS): 3.84 (2.84)	Intervention (3 % HS): F19 M39	Moderate to severe	Intervention (3 % HS): 1.4 (1.41)	NR
	Intervention (5 % HS): 4.02 (2.56)	Intervention (5 % HS): F26 M31		Intervention (5 % HS): 1.56 (1.38)	
	Control: 3.30 (2.43)	Control: F26 M31		Control: 1.88 (1.76)	
Espelt et al. 2012 [25]	NR	Intervention: F24 M26	Moderate	Intervention: 5.8 (2.7)	NR
		Control: F26 M24		Control: 5.47 (2.1)	
Everard et al. 2014 [73]	Intervention: 3.3 (2.6)	Intervention: F69 M73	Severe	Intervention: 4.19 (3.20)	NR
	Control: 3.4 (2.8)	Control: F64 M85		Control: 4.22 (3.52)	
Giudice et al. 2012 [62]	Intervention: 4.8 (2.3)	Intervention: F18 M34	Severe	Intervention: 4.9 (1.3)	Intervention: 6.5 (1.6)
	Control: 4.2 (1.6)	Control:F19 M35		Control: 5.6 (1.6)	Control: 7.7 (1.6)
Kuzik et al. 2007 [20]	Intervention: 4.4 (3.7)	Intervention: F20 M27	Moderate	Intervention: 2.6 (1.9)	NR
	Control: 4.6 (4.7)	Control: F19 M30		Control: 3.5 (2.9)	
Luo et al. 2010 [63]	Intervention: 6.0 (4.3)	NR	Mild to	Intervention: 6 (1.2)	Intervention: 1.5 (0.5)
	Control: 5.6 (4.5)		moderate	Control: 7.4 (1.5)	Control: 2.9 (0.7)
Luo et al. 2011 [64]	Intervention: 5.9 (4.1)	NR	Moderate to severe	Intervention: 4.8 (1.2)	Intervention: 1.7 (0.6)
	Control: 5.8 (4.3)			Control: 6.4 (1.4)	Control: 3.1 (0.7)
Maheshkumar et al. 2013 [67]	NR	NR	Mild to moderate	Intervention: 2.25 (0.89)	NR
				Control: 2.88 (1.76)	
Mandelberg et al. 2003 [18]	Intervention: 3 (1.2)	Intervention: F12 M15	Moderate	Intervention: 3 (1.2)	NR
	Control: 2.6 (1.9)	Control: F9 M15		Control: 4 (1.9)	
Nemsadze et al. 2013 [69]	NR	NR	Mild to moderate	Intervention: 4.4 (1.1)	NR
				Control: 4.9 (1.2)	
Ojha et al. 2014 [72]	Intervention: 8.61 (5.74)	NR	NR	Intervention: 1.87 (0.96)	NR
	Control: 8.51 (4.24)			Control: 1.82 (1.18)	
Ozdogan et al. 2014 [27]	Overall: 7.1 (5.48)	NR	Mild to moderate	NR	NR
Pandit et al. 2013 [66]	NR	NR	Moderate to severe	Intervention: 3.92 (1.72)	NR
				Control: 4.08 (1.90)	
Sharma et al. 2013 [65]	Intervention: 4.93 (4.31)	Intervention: F28 M97	Moderate	Intervention: 2.64 (0.88)	NR
	Control: 4.18 (4.24)	Control: F31 M92		Control: 2.66 (0.93)	
Silver et al. 2014 [71]	Intervention: 3.86 (3.01)	Intervention (3%HS): F31 M62	NR	Intervention: 2.49 (1.64)	NR
	Control: 4.39 (2.95)	Control: F37 M60		Control: 2.47 (1.76)	
Sosa-Bustamante et al. 2014 [26]	NR	NR	Moderate to severe	NR	NR
Tal et al. 2006 [19]	Intervention: 2.8 (1.2)	Intervention: F11 M10	Moderate	Intervention: 2.6 (1.4)	Intervention: 5.35 (1.3)
	Control:2.3 (0.7)	Control: F7 M13		Control: 3.5 (1.7)	Control: 6.45 (1)
Teunissen et al. 2014 [70]	Intervention (3 % HS): 3.6 (5.2)	Intervention (3 % HS): F40 M44	Mild to moderate	Intervention (3 % HS): 3.43 (2.24)	Intervention (3 % HS): 3.87 (3.15)
	Intervention (6 % HS): 3.4 (3.8)	Intervention (6 % HS): F35 M48		Intervention (6 % HS): 3.74 (2.99)	Intervention (6 % HS): 5.16 (4.20)
	Control: 3.6 (5.0)	Control: F31 M49		Control: 2.82 (2.25)	Control: 4.61(5.38)

*F* Female, *M* male, *NR* Not reported, *HS* Hypertonic saline

### Intervention characteristics

All trials administered 3 % hypertonic saline via a nebuliser as the active intervention; three trials were designed with an additional arm using higher concentrations of hypertonic saline at 5 % [27, 68] or 6 % [70]. Fifteen of the eighteen trials stated the amount of hypertonic saline administered which ranged from 3 ml [25, 67] to 4 ml [18–20, 26, 63–66, 70–73] or in one trial 5 ml [68]. Four trials compared hypertonic saline versus normal saline alone [20, 64, 71, 72] or versus standard care [73] with no additional treatments. Where the flow rate was stated (*n* = 10) it ranged between 5 and 10 L/min [18, 19, 62, 65–68, 70, 71, 73]. Investigators in the other trials administered different adjunct treatments alongside hypertonic saline: five trials administered epinephrine/adrenaline [18, 19, 62, 66, 68]; seven administered a β_2_ agonist (salbutamol, albuterol) [25–27, 63, 65, 67, 70]; one trial did not specify any additional medication [69] and five trials did not administer different adjunct treatments alongside hypertonic saline [20, 64, 71–73].

### Comparator characteristics

The majority of trials stated that they administered 0.9 % of normal saline as the control group except one where “oxygen therapy plus best supportive care” was the control [73] or where authors did not state the concentration of normal saline [67, 69]. Eight of the trials gave oxygen therapy in both the intervention and control arms [20, 62, 64, 65, 67, 68, 70, 73], nine trials did not specify whether oxygen therapy was provided [18, 19, 25–27, 63, 69, 71, 72] and one trial stated oxygen therapy was provided in the intervention arm but did not state whether this was provided in the control arm [66]. The flow rate of the nebulisers in the control groups matched those in the intervention arm of each trial.

### Risk of bias

**Fig. 2:**
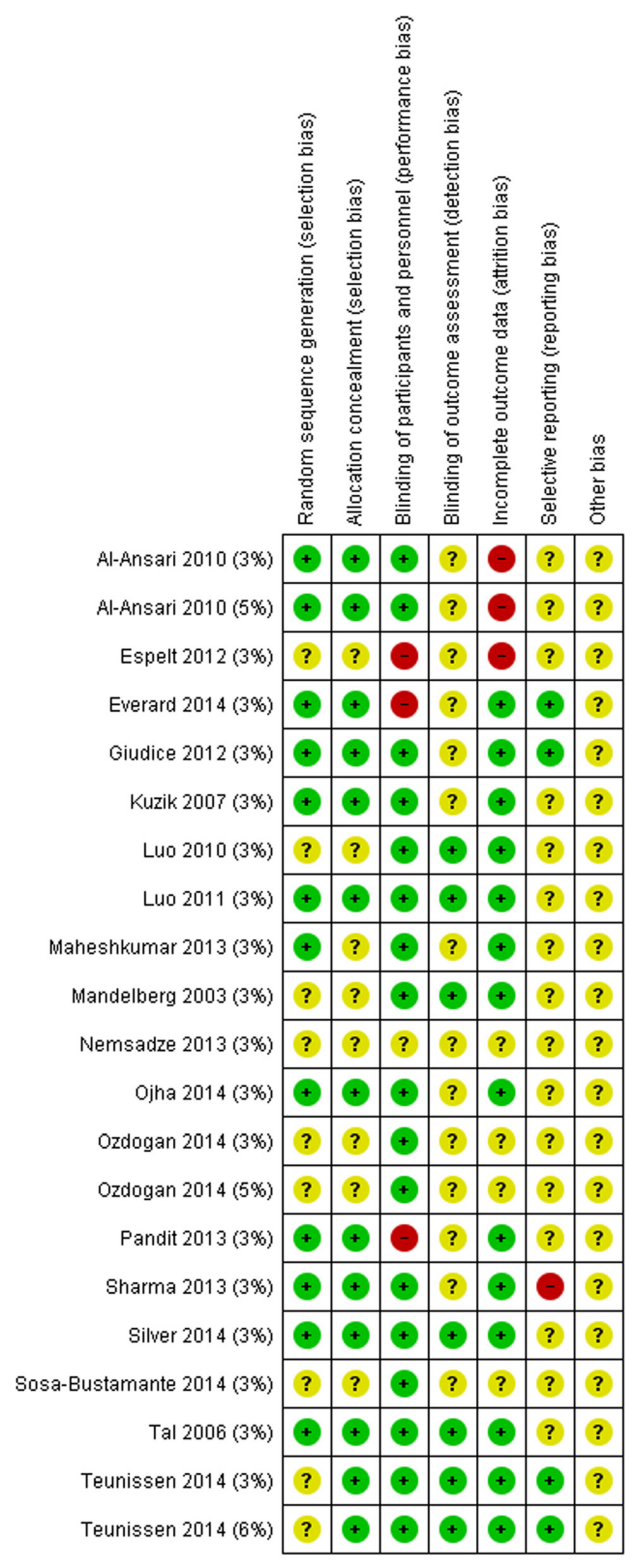
Risk of bias

The risk of bias was variable across studies (Additional file 8 and Fig. 2). Eleven trials had clearly adequate allocation concealment, using pharmacy to prepare and administer solutions [20, 62], sequential identical containers [19, 65, 70], using both these methods [72], a web-based randomisation system [71, 73] or sealed, opaque envelopes [64, 66, 68]. Randomisation was adequately described in eleven trials based on lists generated by computer systems or computer generated random number tables [19, 20, 62, 64–68, 71–73]. Participants and investigators were blinded to the treatment given again in fourteen of the 18 studies; three trials were “open label” [25, 66, 73] and one gave no description of blinding [65]. Blinding of the outcome assessor for the primary outcome was adequately described with the attending physician who made the decision to discharge blinded in five studies [18, 19, 63, 64, 71] and all medical staff, participants and staff blinded in one trial [70]. Participant withdrawal and the use of intention to treat analysis were clearly explained in all but five trials [25–27, 68, 69].

We graded two of these as being at high risk of bias: one provided no explanation regarding the uneven distribution of withdrawals [25] and one did not state which arms the 16 patients were excluded from [68]. The median loss to-follow-up at the time of the primary outcome assessment (LoS) was 8 % (range 0 % [20, 63, 66, 67] to 18 % [25, 72]; twelve studies did not complete an intention to treat analysis, presenting instead an ‘available case analysis’ for only those participants for whom a LoS could be identified [18–20, 25, 62, 64, 65, 68, 70–73]. Three studies completed a full analysis on all participants randomised [63, 66, 67].

### Results and synthesis

**Fig. 3:**
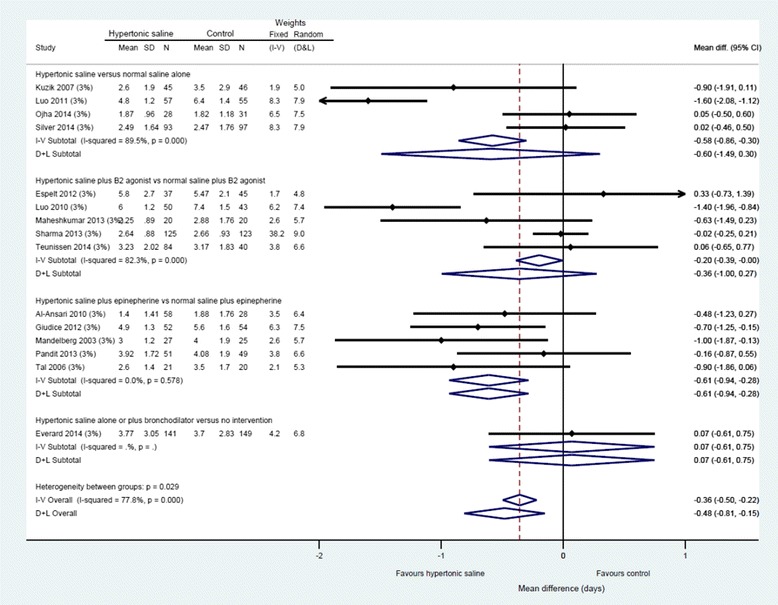
Difference in length of hospital stay by intervention subgroup, 3 % hypertonic saline

**Fig. 4:**
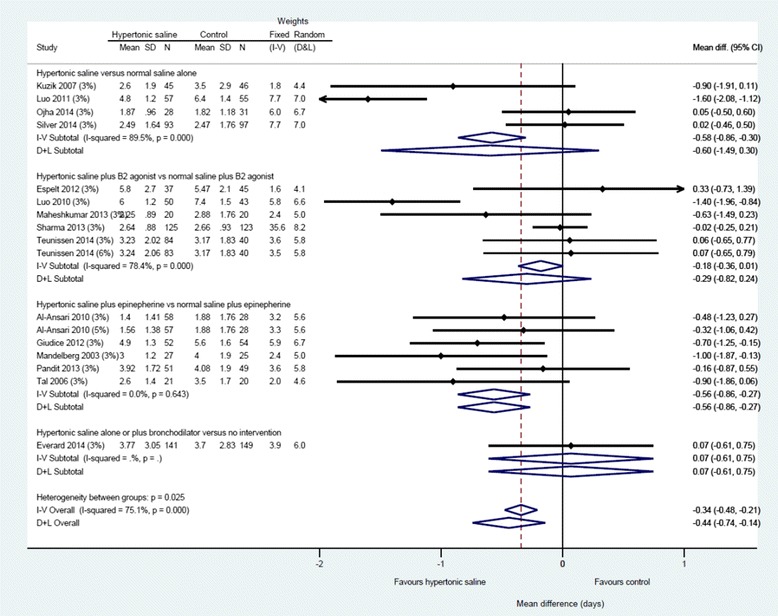
Difference in length of stay by intervention subgroup, all concentrations of hypertonic saline

Data from three studies [26, 27, 69] could not be included in the analysis of the primary outcome (LoS) because it was unclear how many participants were randomised to each study arm or the mean and SD LoS was unavailable; attempts to contact the authors failed. Twelve trials presented available case analyses for the primary outcome, LoS, and the denominators for these trials express the number analysed, rather than the number randomised (Figs. 3 and 4) [18–20, 25, 62, 64, 65, 68, 70–73].

**Fig. 5:**
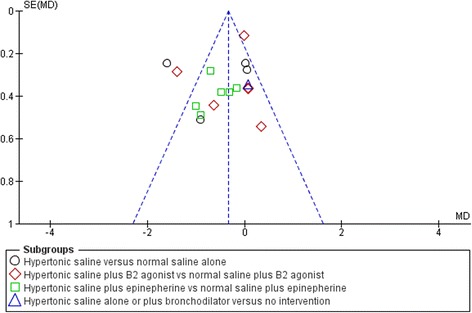
Funnel plot, difference in length of hospital stay (whole group)

Across fifteen studies (*n* = 1922 participants), hypertonic saline, compared with any comparator, reduced mean LoS by approximately a third of a day using a fixed effect approach [mean difference = −0.36, 95 % CI −0.50 to −0.22 days; Fig. 3]. Reflecting the high levels of statistical heterogeneity (I2 = 78 %) a random-effects analysis was undertaken: this gave a pooled effect size of −0.44 (−0.14 to −0.74) The between-trials variance (τ^2^ = 0.27) can be used to derive a prediction interval for the of effect size in a future: this ranged from −1.59 to +0.71. [74] The results are presented in (pre-specified) subgroups based on standard care, but considerable heterogeneity remains even within these. The funnel plot for LoS was not classically symmetrical, but neither was it suggestive of publication bias or small study effects (see Fig. 5).

**Fig. 6:**
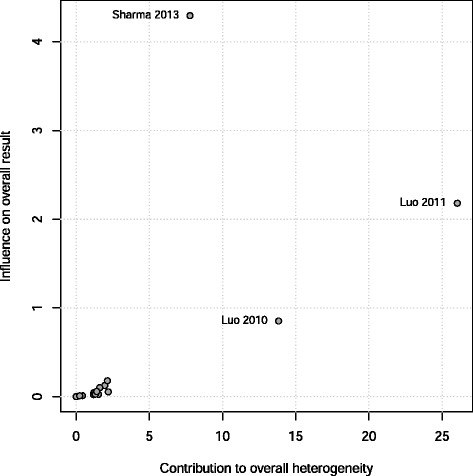
Baujat plot comparing weight to overall heterogeneity

To further explore the heterogeneity we performed sensitivity analyses. Firstly, the contribution of each study to the overall effect size and heterogeneity were assessed graphically by constructing a Baujat plot (Fig. 6) [75]. The figure shows, for each trial, the impact of excluding it from the final analysis (vertical axis), plotted against its contribution to the heterogeneity statistic (horizontal axis). As a general guide, with homogeneity the contribution to heterogeneity (horizontal axis) should range between approx. 0–5 for each study. Two studies are clear outliers, contributing excessively to the heterogeneity [63, 64]; a third contributes to both heterogeneity and effect size, which is to be expected given it contains 38 % of the overall weight [65].

**Fig. 7:**
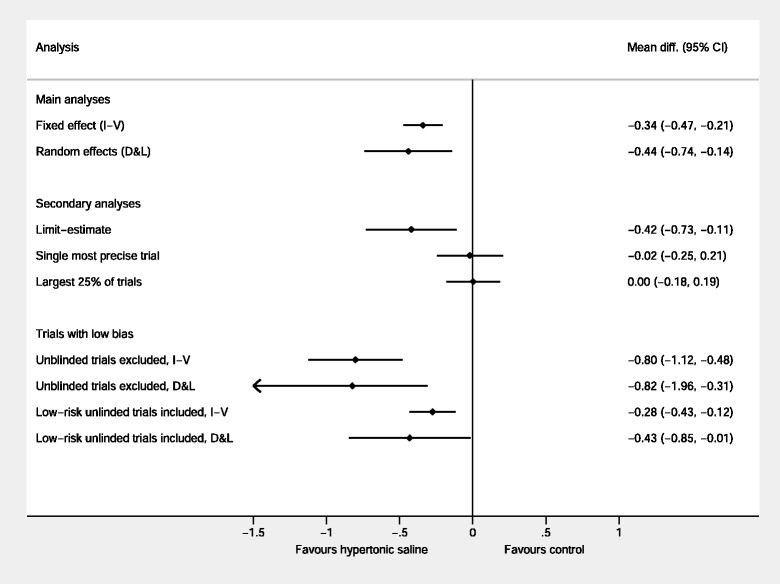
Sensitivity analyses based on precision, size, small study effects and risk of bias

Secondly, we reproduce the approach taken by Dechartres [34] in which the original analyses are compared to those derived from i) the single most precise trial (the trial with the narrowest confidence interval [65]); ii) the pooled average of largest 25 % of trials; iii) the beta0 limit-analysis proposed by Rücker et al. (2011) to control for small-study effects [76]; and iv) restricted to trials at low overall risk of bias. For the latter, we undertook two analyses, the first allowing unblinded trials which were otherwise considered low RoB, and the second limiting to blinded low RoB trials. These secondary analyses are depicted in Fig. 7. Lastly, we repeated the analyses excluding the two outlying trials identified above [63, 64]. The resulting analyses exhibit considerable disparities. The overall results are contradicted by the largest and most precise trials, both of which estimate virtually no difference between the groups. Excluding the two Luo trials greatly reduces the heterogeneity with the overall I^2^ falling from 75 to 23 % and the overall effect size reduced to a more modest (albeit statistically significant) difference of 3.8 h (I-V analysis; 95 % CI 0.2 to 7.2 h) or 5.0 h (D&L analysis; 95 % CI 0.2 to 9.6 h).

**Fig. 8:**
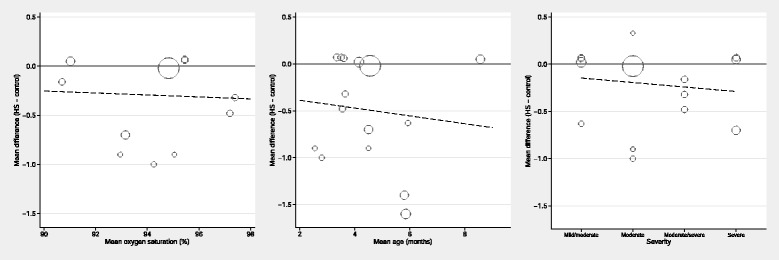
Meta-regression investigating age, baseline oxygen saturation, and severity classification as sources of heterogeneity

Lastly we used meta-regression to explore whether any of the sources of heterogeneity could explain the inconsistency (Fig. 8). Specifically, we assessed the relationship between the effect sizes observed within each trial against its i) mean age; ii) mean baseline oxygen saturation; and iii) severity classification. None of the associations were statistically significant and, more importantly, the residual heterogeneity statistics (ie variation remaining after adjustment) was virtually unchanged, indicating the between-trial variation was not well explained by any of the three characteristics explored.

Five trials contributed to the mean difference in final CSS scores (*n* = 516, MD −1.36 (95 % CI −1.52 to −1.20); we observed no statistical heterogeneity [19, 62–64, 70]. One trial reported one SAE possibly related to the intervention [73], no other studies described intervention related SAE’s.

In an analysis of three studies that reported readmission rate [68, 71, 73] (651 participants), no difference was observed between hypertonic saline and control [RR 0.90, 95 % CI 0.52 to 1.55]; there was no statistical heterogeneity (I2 = 0 %). A decision was made not to attempt a meta-analysis of adverse event data which, where reported, was mainly narrative and not consistently captured across trials. We present a narrative summary in Additional file 9. One study described one SAE, of bradycardia and desaturation during administration of the nebuliser, which had resolved by the following day [73], no other studies described any serious adverse events related to the intervention of hypertonic saline.

## Discussion

### Summary of findings

Overall hypertonic saline was associated with a reduced length of stay, with a pooled difference of −0.36, [95 % CI −0.50 to −0.22]. The evidence for this result is of moderate varies quality (GRADE summary of findings in Table 2). However, our confidence in this estimate is undermined by the excessive heterogeneity of the results (I^2^ = 78 %) and inconsistency between the main and sensitivity analysis in general. Of particular note is that the overall pooled estimate is contradicted by the four largest component trials [65, 70, 71, 73], all of which found no benefit of hypertonic saline. The true impact of hypertonic saline therefore appears to depend greatly on the patient population within which each trial was conducted, and in particular the level of standard care.

**Table 2:** Summary of findings table

	Assumed risk	Corresponding risk	Relative effect (95 % CI)	No of Participants (studies)	Quality of the evidence (GRADE)
Outcomes	Normal saline (+/− adjunct treatment) or oxygen therapy plus best supportive care	Hypertonic saline (+/− adjunct treatment)			
Hypertonic saline versus normal saline alone (days)	The mean length of hospital stay ranged across control groups from 1.82 to 6.4 days	The mean length of hospital stay ranged across hypertonic saline groups from 1.87 to 4.8 days	0.58 (95 % CI −0.86 to −0.30) days	452 (4 inpatient trials)	⊕ ⊕ ⊕ ⊕ high^a^
Hypertonic saline plus B2 agonist vs normal saline plus B2 agonist (days)	The mean length of hospital stay ranged across control groups from 2.66 to 7.4 days	The mean length of hospital stay ranged across hypertonic saline groups from 2.25 to 6 days	0.18 (95 % CI −0.36 to 0.01 days)	710 (5 inpatient trials)	⊕ ⊕ ⊕⊖ moderate^b^
Hypertonic saline plus epinepherine vs normal saline plus epinephrine (days)	The mean length of hospital stay ranged across control groups from 1.88 to 5.6 days	The mean length of hospital stay ranged across hypertonic saline groups from 1.4 to 4.9 days	0.56 (95 % CI −0.86 to −0.27 days)	470 (5 inpatient trials)	⊕ ⊕ ⊕ ⊕ high^c^
Hypertonic saline alone or plus bronchodilator versus no intervention (days)	The mean length of hospital stay for control groups was 3.7 days	The mean length of hospital stay in the hypertonic saline group was 3.7 days	0.07 (95 % CI −0.61 to 0.27 days)	290 (1 inpatient trial)	⊕ ⊕ ⊕⊖ moderate^d^

The basis for the assumed risk (e.g. the median control group risk across studies) is provided in footnotes. The corresponding risk (and its 95 % confidence interval) is based on the assumed risk in the comparison group and the relative effect of the intervention (and its 95 % CI)

*CI* Confidence interval; *RR* Risk Ratio

GRADE Working Group grades of evidence

High quality: Further research is very unlikely to change our confidence in the estimate of effect

Moderate quality: Further research is likely to have an important impact on our confidence in the estimate of effect and may change the estimate

Low quality: Further research is very likely to have an important impact on our confidence in the estimate of effect and is likely to change the estimate

Very low quality: We are very uncertain about the estimate

^a^Substantial heterogeneity; all studies double blinded and generally low risk of bias

^b^Substantial heterogeneity; one study had incomplete outcome data and was un-blinded

^c^No heterogeneity; one study had incomplete outcome data and one was un-blinded

^d^Single study, no blinding

### Strengths and limitations of the findings

High levels of statistical heterogeneity associated with the meta-analysis dominate the results making their interpretation challenging. The addition of concomitant medications may explain some of this heterogeneity across intervention subgroups, with as the largest effect sizes found in trials where hypertonic saline was given alone. Studies were conducted across a number of different healthcare settings with diverse local services, usual care, guidelines (e.g. definition of “fit to discharge”) and disease severity at entry, all of which must contribute to the extensive intra-trial variation observed. That many of the largest trials contributed such little weight to the analysis undermines our confidence in the results. Furthermore, the absence of re-admission rate in the majority of trials may suggest the intervention is not as economically beneficial as the results suggest.

A key strength is the inclusion of 15 of the 18 trials in the meta-analysis of the primary outcome. This facilitated both investigation of publication bias and allowed for subgroup analyses and aggregate data meta-regression, although there was incomplete data in relation to secondary outcomes and trial design features.

One can argue the potential of publication bias based on the uncharacteristic funnel plot shape. Systematic error is not thought to always be caused by application of language restrictions to meta-analyses despite the potential reduction of the precision of pooled estimates [77]. Nonetheless, restriction of trials to only English articles may have altered the precision, effect size, heterogeneity and overall risk of bias.

### Summary of heterogeneity

In undertaking this systematic review it has become apparent that there are a number of semantic, methodological and cultural differences across the studies, all of which impacts on the results obtained and the generalisability of an individual trial’s findings. We propose some of these factors below, and offer an explanation for how these may impact on the interpretation of the review’s findings.

i) The definition of ‘acute bronchiolitis’ differs between countries, and indeed across clinicians in the same institution. Inevitably this diversity was reflected in the description of infants included which variously specified wheeze and or crackles (*n* = 4) [20, 68, 70, 73]; a first episode of wheezing (*n* = 5) [62–66]; “bronchiolitis” (*n* = 4) [19, 67, 71, 72]; or bronchiolitis with a temperature >38C (*n* = 1) [18]; while information was absent in four others [25–27, 69]. The term “wheeze” is itself open to interpretation (and sometimes misinterpretation) within the medical profession [78–82], and may be taken to include children presenting with their first exacerbation of asthma, and manifesting as bronchospasm. The occurrence of this is less common among younger patients, and as a consequence we may have expected the effect size to vary according to the mean age of the study population. Nevertheless, our meta-regression to investigate this was equivocal.

A more immediate explanation is that the impact of HS varied with severity. All patients included in this study met the definition of acute bronchiolitis as used in the UK, Australia and parts of Europe which in summary involves apparent viral infection, signs of lower respiratory tract disease with airflow obstruction manifest by increased work of breathing, hyperinflation of the chest and widespread crackles, with or without intermittent wheeze. Clearly there are considerable differences in setting and in the types of patients included in different studies.

ii) Variation among discharge criteria

The consistency of the outcomes—specifically ‘length of stay’ and ‘fit for discharge’—is self-evidently defined and assessed in very different ways across the studies. Moustgaard et al. suggest that definition of outcomes in trials is a widespread problem[83]. The studies set (sometimes arbitrary) criteria regarding when the patient stay started, including “from study entry, which was within 12 h of admission” (*n* = 2) [20, 62]; from hospital admission (*n* = 3) [65, 68, 73]; or from first dose of study medication (*n* = 2)[70, 71]; information was absent for the remaining 11 studies [18, 19, 25–27, 63, 64, 66, 67, 69, 72]. The reported time to entry into study varied from 3 to 24 h, and generally did not specify whether “entry” corresponded to consent or first treatment. The latter criterion in particular represents a huge proportion of an admission in units with mean stays of 72 h or less. Similarly, discharge was defined and assessed differently across studies. In one study the discharge assessment used a continuous discharge criteria [73],but in at least five others the decision to discharge was made only once a day [18, 19, 63, 65, 66], meaning the time of discharge is effectively a discrete outcome which occurs at intervals of 24 h. Although this inevitably overestimates the real time taken to be fit for discharge, it does so equally for both groups and would be expected to underestimate rather than overestimate the difference between the groups. With this in mind we have no explanation for why the positive studies are based on a once daily clinical assessment.

In the remaining studies the frequency of assessment for discharge was unclear. We present a summary of the discharge criteria in Additional file 10.

The criteria for discharge range from saturating 92 % or greater in air & oral feeding >75 % of usual intake [73] to no respiratory signs or symptoms for the previous 12 h [63, 64]. As may be expected, stricter criteria leads to longer LoS. The criteria that patients should be free of any signs or symptoms is curious as it has been well documented that the symptoms associated with acute bronchiolitis persists for many days or even weeks [73, 84]. Behrendt et al. previously noted a marked variation in length of stay of patients admitted with RSV bronchiolitis with very short admissions [median approx. 72 h] in USA, UK and Northern Europe as compared with significantly longer admissions in Germany and Southern Europe [85] a finding corroborated by more recent trials that have been included herein. These longer admissions were associated with increased co-morbidities such as diarrhoea which may be as a result of nosocomial infection resulting from longer admission times. This cultural difference is again noted with none of the Italian subjects in the study of Giudice being discharged before 72 h, a period beyond the mean ‘length of stay’ in the Dutch, UK and USA study [70, 73, 85]. Finally, the subjects in the Luo studies with mild to moderate [63] bronchiolitis remained in hospital longer than those with more severe disease [64], a finding which is somewhat difficult to explain.

iii) Publication, generalisability and other biases

This difference in practice may also, in large part, explain the differences in observed treatment effects in the large UK, Dutch and USA studies which found no benefit as compared with the apparently large effect observed in other studies [70, 71, 73]. While early indications of a potential benefit may have been attributable to publication bias [86, 87] the positive effects of later large studies may be attributable to study design and cultural effects. It is of note that all the recent studies of hypertonic saline have failed to demonstrate any benefit yet the ‘meta-analysis still appears to favour the treatment. This effect is largely driven by the relatively large studies of Luo et al. and it is likely that this is explicable when considering discharge criteria in more detail (see above).

In summary therefore, there remains considerable heterogeneity which are not germane to being captured and quantified by standard meta-regression tools. Clearly, a large amount of the heterogeneity is driven by two trials from the same team, led by Luo [63, 64], with outlying results, relatively small sample sizes but narrow confidence intervals (around a day, compared with a day-and-a-half in SABRE [73] and the other large northern European study—Teunissen 2014 [70]). The removal of these two studies from the main analysis considerably reduces the effect sizes and statistical significance in the analyses to a more modest (and minimal) impact. Nevertheless, this does not eliminate heterogeneity completely.

Finally, there choice whether to favour a fixed- or random-effects analysis remains open to debate, with strong and apparently compelling proponents on both sides [32, 88–91]. The presence of unexplained heterogeneity goes against the assumption of a single underlying (fixed) effect, and this is commonly taken to justify the random effect model. When the heterogeneity is excessive however, the random effect model has the unfortunate operational characteristic of allocating similar weights to all trials, irrespective of their size and precision. Our decision to pre-specify a fixed-effect as the primary analysis was taken to counter this limitation. That said, we are unable to offer a clinically sensible reason why the largest trial should be allocated only 4 % of the weight in this analysis. Given this, together with the large and unexplained heterogeneity in general, our recommendation is that no single overall summary measure—fixed, random or otherwise—is an adequate reflection of the identified trials. Although we investigated response in relation to dose (3, 5 or 6 %), the studies did not provide data on frequency or duration of HS, which may also have varied across studies.

### Strengths and limitations compared to other reviews

Building on the review conducted by Zhang and colleagues which contained 11 RCTs (*n* = 1090), our review included 15 trials (*n* = 1922) which included three much larger trials which unanimously showed null results [65, 70, 73]. We limited our inclusion criteria to trials of inpatient infants, whereas Zhang et al. also included outpatient and emergency department trials. Al-Ansari has been included in our review despite being included in the emergency department group by Zhang and colleagues, as the length of stay infers that the patients were admitted [68]. Despite this, our meta-analysis included a further 8 trials [25, 65–67, 70–73] which altogether unearthed significantly higher levels of heterogeneity than that stated in the previous Cochrane review. A potential explanation is that we applied no restrictions in terms of dose or way the intervention was administered, and in addition we included data from one unpublished study: Zhang et al. made no statement in regards to these.

Duplication is not without merit—it enables the replicability of methods to be demonstrated, as well as adding weight to or disputing the current evidence base [92–94]. Even when faced with identical data, approaches taken and interpretations made can differ between researchers [95]. A well-defined rationale for any such duplicate review, as required by the PRISMA checklist (though not explicitly) [94], provides transparency regarding overlaps and subsequently, allows for informed debate about its value to the evidence base [95].

### Implications for policy and practice

The disparities between the results of the largest, most precise trials and all the included trials, together with high levels of heterogeneity, mean that neither individual trials nor pooled estimates provide a firm evidence-base for the use of hypertonic saline in inpatient acute bronchiolitis [34]. The refutation of initially large treatment effects in small trials by larger trials stronger is a phenomenon which is observed more widely and should not surprise the reader [96]. For instance, in the treatment of acute bronchiolitis, initial evidence supporting the use of β2-agonists has also been overturned as successive trials have been published [97].

### Further research

Our aggregate level data analysis was unable to identify specific settings and characteristics which influence the effect of HS on LoS. Systematic sensitivity analyses, ideally based on individual patient data and regression analyses are warranted to better understand why hypertonic saline showed substantial benefit in some trials yet none in others [34]. In the absence of this, there is no robust evidence to support the use of hypertonic saline.

## Conclusions

Claims that hypertonic saline achieves small reductions in LoS must be treated with scepticism based on the 15 known trials of HS. The findings appear at best highly dependent on trial design and local policies. We cannot rule out the possibility that inhaled HS offers symptomatic relief but have no data to support or deny this possibility.

## Additional files

Additional file 1:
**PROSPERO registration.** (PDF 109 kb) Additional file 2:
**PRISMA statement.** (DOCX 24 kb) Additional file 3:
**Search strategies.** (DOCX 20 kb) Additional file 4:
**ORBIT classification outcome matrix.** (DOCX 16 kb) Additional file 5:
**Excluded studies at full paper review stage.** (DOCX 20 kb) Additional file 6:
**Study characteristics.** (DOCX 43 kb) Additional file 7:
**Clinical issues.** (DOCX 34 kb) Additional file 8:
**Risk of bias.** (DOCX 21 kb) Additional file 9:
**Adverse events narrative.** (DOCX 22 kb) Additional file 10:
**Discharge criteria.** (DOCX 18 kb)
