# Supplementary material for: Hypertonic saline (HS) for acute bronchiolitis: Systematic review and meta-analysis
Source: BMC Pulm Med. 2015 Nov 23;15:148. doi: 10.1186/s12890-015-0140-x (PMC4657365; doi:10.1186/s12890-015-0140-x)
Supplement: Additional file 1: — PROSPERO registration. (PDF 109 kb) [file 12890_2015_140_MOESM1_ESM.pdf]

PROSPERO International prospective register of systematic reviews

Review title and timescale

1 Review title
Give the working title of the review. This must be in English. Ideally it should state succinctly the interventions or exposures being reviewed and the associated health or social problem being addressed in the review.
Nebulised hypertonic saline solution for acute bronchiolitis in infants: a systematic review and meta-analysis

2 Original language title
For reviews in languages other than English, this field should be used to enter the title in the language of the review. This will be displayed together with the English language title.

3 Anticipated or actual start date
Give the date when the systematic review commenced, or is expected to commence.
01/01/2013

4 Anticipated completion date
Give the date by which the review is expected to be completed.
25/04/2014

5 Stage of review at time of this submission
Indicate the stage of progress of the review by ticking the relevant boxes. Reviews that have progressed beyond the point of completing data extraction at the time of initial registration are not eligible for inclusion in PROSPERO. This field should be updated when any amendments are made to a published record.

The review has not yet started ×

Review stage	Started	Completed
Preliminary searches	No	Yes
Piloting of the study selection process	No	Yes
Formal screening of search results against eligibility criteria	No	Yes
Data extraction	Yes	No
Risk of bias (quality) assessment	Yes	No
Data analysis	Yes	No

Provide any other relevant information about the stage of the review here.

Review team details

6 Named contact
The named contact acts as the guarantor for the accuracy of the information presented in the register record.
Chin Maguire

7 Named contact email
Enter the electronic mail address of the named contact.
c.maguire@sheffield.ac.uk

8 Named contact address
Enter the full postal address for the named contact.
ScHARR, University of Sheffield

9 Named contact phone number
Enter the telephone number for the named contact, including international dialing code.
01142220717

10 Organisational affiliation of the review
Full title of the organisational affiliations for this review, and website address if available. This field may be completed as 'None' if the review is not affiliated to any organisation.
ScHARR, University of Sheffield, 30 Regent Street, Sheffield, S1 4DA

Website address:

11 Review team members and their organisational affiliations
Give the title, first name and last name of all members of the team working directly on the review. Give the organisational affiliations of each member of the review team.

Title	First name	Last name	Affiliation
Mrs	Chin	Maguire	Clinical Trials Unit, University of Sheffield
Miss	Hannah	Cantrill	Clinical Trials Unit, University of Sheffield

12 Funding sources/sponsors
Give details of the individuals, organizations, groups or other legal entities who take responsibility for initiating, managing, sponsoring and/or financing the review. Any unique identification numbers assigned to the review by the individuals or bodies listed should be included.
The results of the systematic review will be presented in the monograph for the " SABRE (hypertonic Saline in Acute Bronchiolitis RCT and Economic evaluation)" Trial funded by the Health Technology Assessment. HTA reference 09/91/22.

13 Conflicts of interest
List any conditions that could lead to actual or perceived undue influence on judgements concerning the main topic investigated in the review.
Are there any actual or potential conflicts of interest?
None known

14 Collaborators
Give the name, affiliation and role of any individuals or organisations who are working on the review but who are not listed as review team members.

Title	First name	Last name	Organisation details
Dr	Daniel	Hind	ScHARR, University of Sheffield

Review methods

15 Review question(s)
State the question(s) to be addressed / review objectives. Please complete a separate box for each question.
How safe and effective is nebulised hypertonic saline solution when used to treat acute bronchiolitis in hospitalised infants (under the age of 2).

16 Searches
Give details of the sources to be searched, and any restrictions (e.g. language or publication period). The full search strategy is not required, but may be supplied as a link or attachment.
The following electronic databases will be searched: Cochrane Central Register of Controlled Trials (CENTRAL); MEDLINE (via Ovid) (1946 to present); EMBASE (1974 to present); Web of Science and Google Scholar (2010 to present). The full search strategy used in each database is attached as a pdf. No restrictions or limits (e.g. age, language, and publication date) will be applied in any of the databases other than Google scholar where a restriction of 2010 onwards will be applied. Trial Registries: Other than electronic database, individual trial registries were searched using the terms “bronchiolitis” and “hypertonic saline”. These included: Clinicaltrials.gov; UK Clinical Trials Gateway (UKCTG); CRD databases (DARE NHS EED, HTA); controlled-trials.com; centrewatch.com and National Research Register (NNR), to identify any unpublished data. Journals: The major journals identified for hand searching are Chest, Paediatrics, and Journal of Paediatrics because these are the journals where the current articles of choice were found. Each of the journals will be searched using the terms "hypertonic saline" and "bronchiolitis". Other searches: The reference lists of all identified and suitable trials will be checked to identify any further trials with a view to obtaining any published data in order to minimise publication bias.

17 URL to search strategy
If you have one, give the link to your search strategy here. Alternatively you can e-mail this to PROSPERO and we will store and link to it.

I give permission for this file to be made publicly available

No

18 Condition or domain being studied

Give a short description of the disease, condition or healthcare domain being studied. This could include health and wellbeing outcomes.

Bronchiolitis (also known as respiratory syncytial virus (RSV)) is a very common respiratory tract infection in young children, most commonly aged between 2-5 months. Acute bronchiolitis results in symptoms of swelling of the airway wall, increased mucous production and an impairment of secretion clearance causing airway obstruction and a combination of gas trapping and ineffective gaseous exchange. Acute bronchiolitis is widely accepted to refer to the first episode of acute wheezing in infants younger than 24 months which may start as an upper respiratory tract viral infection. 1-2% of children diagnosed with bronchiolitis will require hospitalisation. Bronchiolitis is the main cause of hospital admission for respiratory tract infections with recurrent wheezing episodes seen in up to 50% of severely infected children years after their primary diagnosis.

19 Participants/population

Give summary criteria for the participants or populations being studied by the review. The preferred format includes details of both inclusion and exclusion criteria.

Research with children up to the age of 2 years who had been hospitalised as the result of an episode of acute bronchiolitis will be considered for the review. Criteria for inclusion include a first episode of acute wheezing associated with bronchiolitis. Not all cases of bronchiolitis are a result of RSV and as such all cases of bronchiolitis regardless of organism will be included.

20 Intervention(s), exposure(s)

Give full and clear descriptions of the nature of the interventions or the exposures to be reviewed

The intervention under consideration is nebulised hypertonic saline with or without an adjunct treatment given versus normal saline or no intervention (control). These can be summarised in the following groups: 1. Nebulised hypertonic saline alone vs normal saline 2. Nebulised hypertonic saline plus a bronchodilator (e.g. salbutamol) vs. normal saline 3. Nebulised hypertonic saline plus a bronchodilator (e.g. salbutamol) vs. normal saline plus same bronchodilator 4. Nebulised hypertonic saline alone or plus a bronchodilator (e.g. salbutamol) vs. no intervention No restrictions will be applied in terms of the concentration, dose or the way the intervention (hypertonic saline) or control (normal saline with or without adjunct treatment) is administered in the trials.

21 Comparator(s)/control

Where relevant, give details of the alternatives against which the main subject/topic of the review will be compared (e.g. another intervention or a non-exposed control group).

Please see 'Intervention(s), exposure(s)' above for outline of comparators used in the review.

22 Types of study to be included initially

Give details of the study designs to be included in the review. If there are no restrictions on the types of study design eligible for inclusion, this should be stated.

Published and unpublished, randomised (RCTs) and quasi randomised trials; cohort and other observational studies will be excluded. Only trials which have completed recruitment will be included in the review. No language or publication restrictions will be applied at the search stage however only those trials published in English language will be included in the review.

23 Context

Give summary details of the setting and other relevant characteristics which help define the inclusion or exclusion criteria.

Studies will not be excluded based on the outcomes they measure; only the population and intervention will be used to screen trials for the review. A systematic review by Zhang et al conducted in 2010 suggests that hypertonic saline results in improved clinical outcomes for infants with viral bronchiolitis. The review looked at children in a number of different settings (hospitalised, outpatients and those in the emergency department). This review will update the systematic review conducted in 2010 (with respect to hospitalised infants only) to incorporate both new published and unpublished data from recent clinical trials conducted since 2010.

24 Primary outcome(s)

Give the most important outcomes.

The primary objective of this review is to determine whether nebulised hypertonic saline results in benefits to hospitalised children in terms of reducing the length of hospital stay or time taken to be ready for discharge.

Give information on timing and effect measures, as appropriate.

A 'summary of findings' table will be included in the results section. For any dichotomous outcomes results will be

expressed as risk ratios (RR) and 95% confidence interval (CI). For any continuous outcomes the mean difference (MD) and 95% CI will be used.

25 Secondary outcomes

List any additional outcomes that will be addressed. If there are no secondary outcomes enter None.

Secondary outcomes of interest include: rate of readmission to hospital; any adverse events however described but particularly tachycardia, hypertension, pallor, tremor, nausea, vomiting and acute urinary retention; and final Clinical Severity Scores (CSS).

Give information on timing and effect measures, as appropriate.

26 Data extraction, (selection and coding)

Give the procedure for selecting studies for the review and extracting data, including the number of researchers involved and how discrepancies will be resolved. List the data to be extracted.

The titles and abstracts of all the studies identified by the search will be performed. The full articles of any studies that appear to meet the inclusion criteria or those where it is unclear, or where there is insufficient information to make a decision for their inclusion, will be retrieved. Papers that do not meet the inclusion criteria will be excluded. Data will be extracted onto a standardised data extraction form to both assess the methodological quality of the studies and retrieve outcome data. Key data to be extracted include: 1) study overview (country, year) 2) participant characteristics (age, number randomised, baseline imbalances assessed by the authors in trials, withdrawals, % allocated completing follow up, illness severity, eligibility) 3) intervention and control group details (number randomised in each group, intervention details: duration, delivery, other drugs and compliance) 4) Outcomes data: a) for continuous outcomes: LoS (mean LoS, SD and number of patients in each group, measured by who); CSS (mean final CSS, SD, number of patients for both groups). Principal summary measure is weighted mean difference in LoS. b) qualitative AE data as available

27 Risk of bias (quality) assessment

State whether and how risk of bias will be assessed, how the quality of individual studies will be assessed, and whether and how this will influence the planned synthesis.

Risk of bias assessment will be performed on the extracted data based on the Cochrane Collaboration's recommendations to assess the quality of the studies. Data will be summarised in the "risk of bias" tables in the results of the review. Trials will be graded on their quality as "A" low risk of bias, "B" high risk of bias or "C" risk of bias unclear. Where a "C" grading is given, every effort will be made to obtain further information to categorise the trial by contacting the trial authors within the specified time frame of this piece of work. The funnel plot method will be used to investigate whether publication bias is present if sufficient studies are included in the meta-analysis. Any unpublished results that are obtained will be incorporated. Risk of bias assessment data items will include: sequence generation; allocation concealment; blinding (outcome and personnel); incomplete outcome data and selective reporting Data will be input into RevMan to generate summary statistics.

28 Strategy for data synthesis

Give the planned general approach to be used, for example whether the data to be used will be aggregate or at the level of individual participants, and whether a quantitative or narrative (descriptive) synthesis is planned. Where appropriate a brief outline of analytic approach should be given.

The primary outcome data collected are the Length of Hospital Stay (LoS) SD and number of participants in both intervention and control groups. A fixed effect model will be used to analyse these continuous data based on the assumption that LoS outcome would estimate the same effect size in each of the studies (Borenstein, 2007). A weighted mean difference and associated 95% confidence interval will be calculated (via RevMan) in order to generate a forest plot. The fixed effect model allows the studies to be weighted depending on their sample size; the greater the sample size, the greater the weight assigned to the trial. The secondary outcomes of interest includes adverse events (however reported), rates of hospital re-admission and final CSS. Data on adverse events will be collected (however these are defined) and a descriptive narrative of the results will be undertaken. These form part of the qualitative synthesis of the results.

29 Analysis of subgroups or subsets

Give any planned exploration of subgroups or subsets within the review. 'None planned' is a valid response if no subgroup analyses are planned.

None planned.

Review general information

30 Type of review

Select the type of review from the drop down list.
Intervention

31 Language
Select the language(s) in which the review is being written and will be made available, from the drop down list. Use the control key to select more than one language.
English

Will a summary/abstract be made available in English?
No

32 Country
Select the country in which the review is being carried out from the drop down list. For multi-national collaborations select all the countries involved. Use the control key to select more than one country.
England

33 Other registration details
List places where the systematic review title or protocol is registered (such as with he Campbell Collaboration, or The Joanna Briggs Institute). The name of the organisation and any unique identification number assigned to the review by that organization should be included.
None.

34 Reference and/or URL for published protocol
Give the citation for the published protocol, if there is one.
None available

Give the link to the published protocol, if there is one. This may be to an external site or to a protocol deposited with CRD in pdf format.

I give permission for this file to be made publicly available
No

35 Dissemination plans
Give brief details of plans for communicating essential messages from the review to the appropriate audiences.
The review will form part of the HTA monograph for the Sabre Clinical Trial.

Do you intend to publish the review on completion?
Yes

36 Keywords
Give words or phrases that best describe the review. (One word per box, create a new box for each term)
hypertonic saline

bronchiolitis

infants

37 Details of any existing review of the same topic by the same authors
Give details of earlier versions of the systematic review if an update of an existing review is being registered, including full bibliographic reference if possible.
None

38 Current review status
Review status should be updated when the review is completed and when it is published.
Ongoing

39 Any additional information

Provide any further information the review team consider relevant to the registration of the review.

40 Details of final report/publication(s)

This field should be left empty until details of the completed review are available.
Give the full citation for the final report or publication of the systematic review.
Give the URL where available.
