# Supplementary material for: Hypertonic saline (HS) for acute bronchiolitis: Systematic review and meta-analysis
Source: BMC Pulm Med. 2015 Nov 23;15:148. doi: 10.1186/s12890-015-0140-x (PMC4657365; doi:10.1186/s12890-015-0140-x)
Supplement: Additional file 2: — PRISMA statement. (DOCX 24 kb) [file 12890_2015_140_MOESM2_ESM.docx]

**Discharge criteria**

| **Study** | **Primary outcome** | **LoS starting time point** | **End of period assessed as being ‘LOS’** | **Clinical Score**  **For discharge** | **O_2_ Stats in air**  **For discharge** | **Clinical grounds by attending physician** | **Frequency of assessment for discharge** |
| --- | --- | --- | --- | --- | --- | --- | --- |
| Al-Ansari et al 2010 [67] | Wang clinical score pre-nebulisation at 48hrs, most discharged by 48 hrs  [LOS not stated as outcome] | Admission to ED short stay ward | Time to leaving short stay ward  [affected by medical and social factors] | *<4 | >94% | Treating physician determined no need supplemental oxygen, feeding adequately, minimal or absent wheezing or crackles | NR |
| Espelt et al 2012 [25] | ‘LOS’ | NR – Clinical trials.gov only | NR – Clinical trials.gov only | NR – Clinical trials.gov only | NR – Clinical trials.gov only | NR – Clinical trials.gov only | NR – Clinical trials.gov only |
| Everard et al 2014 [72] | Time to fit for discharge | Admission | Met protocol defined fit for discharge criteria | NR | >92% in air for 6hours | NR | Data recorded hourly, participants could be discharged every hour |
| Giudice et al 2012 [61] | ‘LOS’ | From study entry which was within 12 hours of admission to hospital | Discharged on the basis of clinical grounds by attending physician | NR | NR | Discharged on the basis of clinical grounds by attending physician | NR  Potentially daily – clinical scoring only every 24 hours, no participant discharged prior to 72 hours. |
| Kuzik et al 2007 [20] | ‘LOS’ | From study entry which was within 12 hours of admission to hospital. | Met protocol defined fit for discharge criteria or discharge on independent clinical grounds. | *<4 | >95% for 4 hours | Met protocol discharge criteria (clinical score and sats or on “clinical ground’) | NR  Potentially daily – clinical scoring and sats ‘at least’ every 24 hrs. |
| Luo et al 2010 [62] | Effect on clinical score and ‘LOS’ | NR  [first treatment within 12 hours] | NR | NR | NR | No respiratory signs or symptoms in previous 12 hours | Morning ward round |
| Luo et al 2011 [63] | ‘LOS’ | NR  [assessed for eligibility within 2 hours of admission] | NR | NR | NR | No respiratory signs or symptoms in previous 12 hours | NR  Potentially morning ward rounds – conducted in same centre as Luo et al 2010 |
| Maheshkumar et al 2013 [66] | Improvement in clinical score and ‘LOS’ | NR  [all participants enrolled within 24 hours of admission] | NR  [‘discharged from study’] | NR | >96% | No respiratory distress and tolerating oral feeds well | NR  Potentially daily – clinical scoring only every 24 hours |
| Mandelberg et al 2003 [18] | Severity scores and ‘LOS’ | NR  [enrolled within 24 hrs of admission] | NR | NR | NR | Discharged on the basis of clinical grounds by attending physician | Morning ward rounds |
| Nemsadze et al 2013 [68] | Change in clinical score  [‘LOS’ secondary] | NR – Abstract only | NR – Abstract only | NR – Abstract only | NR – Abstract only | NR – Abstract only | NR – Abstract only |
| Ojha et al 2014 [71] | ‘LOS’ and clinical score | NR  [enrolled within 24 hrs of admission to ward] | NR  [‘time of discharge’ suggests this is when clinical criteria for discharge is met] | *<4 | >94% for 4 hours | Minimal or absent wheezing required for discharge | NR |
| Ozdogan et al 2014 [27] | Change in clinical score, ‘LOS’ | NR - Abstract only | NR - Abstract only | NR - Abstract only | NR - Abstract only | NR - Abstract only | NR - Abstract only |
| Pandit et al 2013 [65] | ‘LOS’ | NR | NR | NR | NR | Respiratory Rate <60/min and no wheeze or crackles | Once a day |
| Sharma et al 2013 [64] | ‘LOS’ | From hospital admission | *<3 | NR | >92% | Feeding well orally | Participants examined daily, clinical score every 12 hours |
| Silver et al 2014 [70] | ‘LOS’ and  readmission for bronchiolitis within 30 days | Time from first study treatment | “Time of discharge order” | NR | NR | NR | NR |
| Sosa-Bustamante et al 2014 [26] | Clinical score [‘LOS’ secondary] | NR | NR | NR | NR | NR | NR |
| Tal et al 2006 [19] | Duration of admission and change in clinical score | NR  [enrolled within 24 hrs of admission] | NR | NR | NR | Decision to discharge based on clinical ground alone. | Moring ward round |
| Teunissen et al 2014 [69] | ‘LOS’ | 1^st^ dose of study medication | Clinical decision to discharge | NR | >93% | NR | NR |
| * Respiratory Distress Assessment Instrument, **Clinical scoring of respiratory distress | | | | | | | |
| LOS-Length of stay  HS-Hypertonic saline  NS-Normal saline  Sats-O_2_ Saturations  NR-Not reported | | | | | | | |
