# Supplementary material for: Hypertonic saline (HS) for acute bronchiolitis: Systematic review and meta-analysis
Source: BMC Pulm Med. 2015 Nov 23;15:148. doi: 10.1186/s12890-015-0140-x (PMC4657365; doi:10.1186/s12890-015-0140-x)
Supplement: Additional file 4: — ORBIT classification outcome matrix. (DOCX 16 kb) [file 12890_2015_140_MOESM4_ESM.docx]

## Search strategies

**Medline (via Ovid)**

Medline was searched from 1946 (inception) to present. No limits (e.g. dates, age) or restrictions were applied.

Search date: January 2015.

1. Bronchiolitis/ (2310 results)
2. Bronchiolitis.mp. [mp = title, abstract, original title, name of substance word, subject heading word, keyword heading word, protocol supplementary concept, rare disease supplementary concept, unique identifier] (9842 results)
3. 1 OR 2 (9842 results)
4. Humans/ (13622085 results)
5. Humans.mp. [mp = title, abstract, original title, name of substance word, subject heading word, keyword heading word, protocol supplementary concept, rare disease supplementary concept, unique identifier] (13701574 results)
6. 4 OR 5 (13701574 results)
7. 3 AND 6 (8487 results)
8. Aerosols/ or “Nebulizers and Vaporizers”/ (30740 results)
9. nebulize$.mp. [mp = title, abstract, original title, name of substance word, subject heading word, keyword heading word, protocol supplementary concept, rare disease supplementary concept, unique identifier] (11059 results)
10. 8 OR 9 (33444 results)
11. 7 AND 10 (238 results)
12. Sodium Chloride/ or Saline Solution, Hypertonic/ (54351 results)
13. Hypertonic saline.mp. [mp = title, abstract, original title, name of substance word, subject heading word, keyword heading word, protocol supplementary concept, rare disease supplementary concept, unique identifier] (4987 results)
14. 12 OR 13 (56498 results)
15. **11 AND 14 (43 results)**

**Embase**

Embase was searched from 1974 (inception) to present. No limits (e.g. dates, age) or restrictions were applied.

Search date: January 2015.

1. Bronchiolitis/ (9588 results)
2. Bronchiolitis.tw. (11033 results)
3. 1 OR 2 (14695 results)
4. Human/ (15229989 results)
5. Human.mp. [mp = title, abstract, subject headings, heading word, drug trade name, original title, device manufacturer, drug manufacturer, device trade name, keyword] (15981086 results)
6. 4 OR 5 (15981086 results)
7. 3 AND 6 (12665 results)
8. Aerosol/ or nebulizer/ or nebulization/ (50225 results)
9. Nebulize$.mp. [mp = title, abstract, subject headings, heading word, drug trade name, original title, device manufacturer, drug manufacturer, device trade name, keyword] (11285 results)
10. 8 OR 9 (53556 results)
11. 7 AND 10 (413 results)
12. Sodium chloride/ (134876 results)
13. Hypertonic saline.mp [mp = title, abstract, subject headings, heading word, drug trade name, original title, device manufacturer, drug manufacturer, device trade name, keyword] (6139 results)
14. 12 OR 13 (136836 results)
15. **11 AND 14 (102 results)**

**Cochrane Database (CENTRAL)**

CENTRAL was searched from inception to present. No limits (e.g. dates, age) or restrictions were applied.

Search date: January 2015.

1. MeSH descriptor: [Bronchiolitis] (279 results)
2. Bronchiolitis (749 results)
3. 1 OR 2 (750 results)
4. MeSH descriptor: [Humans] (1100 results)
5. Human (504536 results)
6. 4 OR 5 (504536 results)
7. 3 AND 6 (444 results)
8. MeSH descriptor: [Nebulizers and Vaporizers] (1889 results)
9. Nebulize$ (13 results)
10. MeSH descriptor: [Aerosols] (2107 results)
11. 8 OR 9 OR 10 (3713 results)
12. 7 AND 11 (78 results)
13. MeSH descriptor: [Saline Solution, Hypertonic] (404 results)
14. Hypertonic saline (949 results)
15. 13 OR 14 (949 results)
16. **12 AND 15 (18 results)**

**Google Scholar**

Bronchiolitis AND hypertonic saline performed from 2010 to present. 915 articles were found and reviewed.

**Web of Science**

Bronchiolitis AND hypertonic saline from 2010 to present. 81 articles were found and reviewed.
