# Supplementary material for: Hypertonic saline (HS) for acute bronchiolitis: Systematic review and meta-analysis
Source: BMC Pulm Med. 2015 Nov 23;15:148. doi: 10.1186/s12890-015-0140-x (PMC4657365; doi:10.1186/s12890-015-0140-x)
Supplement: Additional file 5: — Excluded studies at full paper review stage. (DOCX 20 kb) [file 12890_2015_140_MOESM5_ESM.docx]

**ORBIT classification outcome matrix**

| Outcome Matrix | |  |  |  |  |  |  |  |
| --- | --- | --- | --- | --- | --- | --- | --- | --- |
| Main Author | Primary outcome (LoS) | ORBIT Classification | Adverse events | ORBIT Classification | Hospital re-admission rate | ORBIT Classification | Final CSS score | ORBIT Classification |
| Al-Ansari et al 2010 [67] | x | n/a | x | n/a | x | n/a | o | I, No risk |
| Espelt et al 2012 [25] | x | n/a | o | F, Low risk | o | I, No risk | o | F, Low risk |
| Everard et al 2014 [72] | x | n/a | x | n/a | x | n/a | o | I, No risk |
| Giudice et al 2012 [61] | x | n/a | o | F, Low risk | o | I, No risk | x | n/a |
| Kuzik et al 2007 [20] | x | n/a | x | n/a | o | I, No risk | Partial (reported on different scale) | F, Low risk |
| Luo et al 2010 [62] | x | n/a | x | n/a | o | I, No risk | x | n/a |
| Luo et al 2011 [63] | x | n/a | x | n/a | o | I, No risk | x | n/a |
| Maheshkumar et al 2013 [66] | x | n/a | Partial (concluded "it's safe") | F, Low risk | o | I, No risk | Partial (final score not provided) | C, Low risk |
| Mandelberg et al 2003 [18] | x | n/a | x | n/a | o | I, No risk | Partial (only % decrease provided) | F, Low risk |
| Nemsadze et al 2013 [68] | Partial | C, Low risk (abstract only) | o | F, low risk (abstract only) | o | H, Low risk (abstract only) | o | H, Low risk (abstract only) |
| Ojha et al 2014 [71] | x | n/a | o | E, High risk | o | I, No risk | o | I, No risk |
| Ozdogan et al 2014 [27] | Partial | C, Low risk (abstract only) | o | F, Low risk (abstract only) | o | H, Low risk (abstract only) | o | I, No risk |
| Pandit et al 2013 [65] | x | n/a | x | n/a | o | I, No risk | o | I, No risk |
| Sharma et al 2013 [64] | x | n/a | x | n/a | o | I, No risk | o | C, Low risk |
| Silver et al 2014 [70] | x | n/a | x | F, Low risk (clinical trials.gov only) | x | n/a | o | I, No risk |
| Sosa-Bustamante et al 2014 [26] | O | F, Low risk (clinical trials.gov only) | o | F, Low risk (clinical trials.gov only) | o | H, Low risk (clinical trials.gov only) | o | I, No risk |
| Tal et al 2006 [19] | x | n/a | x | n/a | o | I, No risk | x | n/a |
| Teunissen et al 2014 [69] | x | n/a | x | n/a | o | I, No risk | x | n/a |
| X | Full reporting of results for comparison | | | | | | | |
| O | No reporting of results | | | | | | | |
| Partial | Partial reporting (e.g. only p value) | | | | | | | |
| C | States outcome analysed but insufficient data presented to be included in meta-analysis or to be considered to be fully tabulated. | | | | | | | |
| E | Clear that outcome was measured but not necessarily analysed e.g. safety reporting | | | | | | | |
| F | Clear that outcome was measured but not necessarily analysed. | | | | | | | |
| I | Outcome not measured or analysed | | | | | | | |
| H | Not mentioned but clinical judgment says unlikely to have been measured. | | | | | | | |
