# Supplementary material for: Hypertonic saline (HS) for acute bronchiolitis: Systematic review and meta-analysis
Source: BMC Pulm Med. 2015 Nov 23;15:148. doi: 10.1186/s12890-015-0140-x (PMC4657365; doi:10.1186/s12890-015-0140-x)
Supplement: Additional file 6: — Study characteristics. (DOCX 43 kb) [file 12890_2015_140_MOESM6_ESM.docx]

Excluded studies at full paper review stage

| **Studies excluded at full paper review** | **Reason for exclusion** |
| --- | --- |
| Ipek et al 2011 [1] | Wrong Setting |
| Anil et al 2010 [2] | Wrong Setting |
| Grewal et al 2009 [3] | Wrong Setting |
| Kuzik et al 2010 [4] | Wrong Setting |
| Sezer et al 2010 [5] | Wrong Setting |
| Jacobs et al 2014 [6] | Wrong Setting |
| Wu et al 2014 [7] | Wrong Setting |
| Park et al 2005 [8] | Not in English |
| Zheng et al 2012 [9] | Not in English |
| Gutpa et al 2012 [10] | Wrong population or intervention |
| Khashabi et al 2005 [11] | Wrong population or intervention |
| Lines et al 1992 [12] | Wrong population or intervention |
| Milner et al 1995 [13] | Wrong population or intervention |
| Patel et al 2002 [14] | Wrong population or intervention |
| Tinsa et al 2009 [15] | Wrong population or intervention |
| Wainwright et al 2003 [16] | Wrong population or intervention |
| Postiaux et al 2011 [17] | Wrong population or intervention |
| Hariprakash et al 2003 [18] | Wrong population or intervention |
| Sarrell et al 2002 [19] | Wrong population or intervention |
| Chowdhury et al 1995 [20] | Wrong population or intervention |
| Nenna et al 2009 [21] | Wrong population or intervention |
| Bertrand et al 2001 [22] | Wrong population or intervention |
| Nenna et al 2013 [23] | Wrong population or intervention |
| Bueno Campaña et al 2014 [24] | Wrong population or intervention |
| Mandelberg et al 2010 [25] | Not a controlled trial |
| Principi et al 2011 [26] | Not a controlled trial |
| Hom et al 2011 [27] | Not a controlled trial |
| Sauvaget et al 2012 [28] | Not a controlled trial |

**References**

1. Ipek IO, Yalcin EU, Sezer RG, Bozaykut A: **The efficacy of nebulized salbutamol, hypertonic saline and salbutamol/hypertonic saline combination in moderate bronchiolitis.** *Pulm Pharmacol Ther* 2011, **24**:633–7.

2. Anil AB, Anil M, Saglam AB, Cetin N, Bal A, Aksu N: **High volume normal saline alone is as effective as nebulized salbutamol-normal saline, epinephrine-normal saline, and 3% saline in mild bronchiolitis.** *Pediatr Pulmonol* 2010, **45**:41–7.

3. Grewal S, Ali S, McConnell DW, Vandermeer B, Klassen TP: **A randomized trial of nebulized 3% hypertonic saline with epinephrine in the treatment of acute bronchiolitis in the emergency department**. *Arch Pediatr Adolesc Med* 2009, **163**:1007–1012.

4. Kuzik BA, Flavin MP, Kent S, Zielinski D, Kwan CW, Adeleye A, Vegsund BC, Rossi C: **Effect of inhaled hypertonic saline on hospital admission rate in children with viral bronchiolitis: a randomized trial**. *CJEM Can Emerg Med care = JCMU J Can soins.m�dicaux.d’urgence* 2010, **12**:477–484.

5. Sezer GR, Bozaykut A, Ipek IO, Uyur E, Seren PL, Paketci C: **The efficacy of nebulized salbutamol, hypertonic saline and salbutamol / hypertonic saline combination in first bronchiolitis attack.** *Acta Paediatr* 2010, **99**:153.

6. Jacobs JD, Foster M, Wan J, Pershad J: **7% Hypertonic saline in acute bronchiolitis: a randomized controlled trial.** *Pediatrics* 2014, **133**:e8–13.

7. **Nebulized Hypertonic Saline for Bronchiolitis: A Randomized Clinical Trial.**

8. Park JY, Jeong YM, Jeong SJ, Seo SS: **The efficacy of nebulized 3 percent hypertonic saline solution and fenoterol in infants with bronchiolitis**. *Korean J Pediatr* 2005, **48**:518–522.

9. Zheng W, Li L, Chengfung H, Yunmei H, Wei L: **The effects of inhalation of the 3% hypertonic saline solution with ambroxol hydrochloride in the treatment of 43 bronchiolitis patients**. *J Pediatr Pharm* 2012, **18**.

10. Gupta N, Puliyel A, Manchanda A, Puliyel J: **Nebulized hypertonic-saline vs epinephrine for bronchiolitis: Proof of concept study of cumulative sum (CUSUM) analysis**. *Indian Pediatr* 2012, **49**:July.

11. Khashabi J, Salari LS, Karamiyar M, Mussavi H: **Comparison of the efficacy of nebulized L-epinephrine, salbutamol and normal saline in acute bronchiolitis: A randomized clinical trial**. *Med J Islam Repub Iran* 2005, **19**:2005.

12. Lines DR, Bates ML, Rechtman AR, Sammartino LP: **Efficacy of nebulised ipratropium bromide in acute bronchiolitis**. *Pediatr Rev Commun* 1992, **6**:1992.

13. Milner A: **The role of anticholinergic in acute bronchiolitis in infancy. [French]**. *Arch Pediatr* 1995, **2**:1995.

14. Patel H, Platt RW, Pekeles GS, Ducharme FM: **A randomized, controlled trial of the effectiveness of nebulized therapy with epinephrine compared with albuterol and saline in infants hospitalized for acute viral bronchiolitis**. *J Pediatr* 2002, **141**:818–824.

15. Tinsa F, Rhouma AB, Ghaffari H, Boussetta K, Zouari B, Brini I, Karboul L, Souid M, Bousnina S: **A randomized, controlled trial of nebulized terbutaline in the first acute bronchiolitis in infant less than 12 months old**. *Tunisie Medicale* 2009, **87**:March.

16. Wainwright C, Altamirano L, Cheney M, Cheney J, Barber S, Price D, Moloney S, Kimberley A, Woolfield N, Cadzow S, Fiumara F, Wilson P, Mego S, VandeVelde D, Sanders S, O’Rourke P, Francis P: **A multicenter, randomized, double-blind, controlled trial of nebulized epinephrine in infants with acute bronchiolitis**. *N Engl J Med* 2003, **349**:27–35.

17. Postiaux G, Louis J, Labasse HC, Gerroldt J, Kotik AC, Lemuhot A, Patte C: **Evaluation of an alternative chest physiotherapy method in infants with respiratory syncytial virus bronchiolitis**. *Respir Care* 2011, **56**:989–994.

18. Hariprakash S, Alexander J, Carroll W, Ramesh P, Randell T, Turnbull F, Lenney W: **Randomized controlled trial of nebulized adrenaline in acute bronchiolitis**. *Pediatr Allergy Immunol* 2003, **14**:134–139.

19. Sarrell EM, Tal G, Witzling M, Someck E, Houri S, Cohen HA, Mandelberg A: **Nebulized 3% hypertonic saline solution treatment in ambulatory children with viral bronchiolitis decreases symptoms**. *Chest* 2002, **122**:2015–2020.

20. Chowdhury D, al HM, Khalil M, al-Frayh AS, Chowdhury S, Ramia S: **The role of bronchodilators in the management of bronchiolitis: a clinical trial**. *Ann Trop Paediatr* 1995, **15**:77–84.

21. Nenna R, Tromba V, Berardi R, De AD, Papoff P, Sabbatino G, Moretti C, Midulla F: **Recombinant human deoxyribonuclease treatment in hospital management of infants with moderate-severe bronchiolitis**. *Eur J Inflamm* 2009, **7**:September.

22. Bertrand P, Aranibar H, Castro E, Sanchez I: **Efficacy of nebulized epinephrine versus salbutamol in hospitalized infants with bronchiolitis**. *Pediatr Pulmonol* 2001, **31**:2001.

23. Nenna R, Papoff P, Moretti C, De Angelis D, Battaglia M, Papasso S, Bernabucci M, Cangiano G, Petrarca L, Salvadei S, Nicolai A, Ferrara M, Bonci E, Midulla F: **Seven percent hypertonic saline-0.1% hyaluronic acid in infants with mild-to-moderate bronchiolitis.** *Pediatr Pulmonol* 2013.

24. Bueno Campaña M, Olivares Ortiz J, Notario Muñoz C, Rupérez Lucas M, Fernández Rincón A, Patiño Hernández O, Calvo Rey C: **High flow therapy versus hypertonic saline in bronchiolitis: randomised controlled trial.** *Arch Dis Child* 2014:1–5.

25. Mandelberg A: **Hypertonic saline in the treatment of acute bronchiolitis in the emergency department**. *Arch Pediatr Adolesc Med* 2010, **164**:395–397.

26. Principi T, Komar L: **A critical review of “a randomized trial of nebulized 3% hypertonic saline with epinephrine in the treatment of acute bronchiolitis in the emergency department.”** *J Popul Ther Clin Pharmacol* 2011, **18**:e273–e274.

27. Hom J, Fernandes RM: **When should nebulized hypertonic saline solution be used in the treatment of bronchiolitis?**. *Paediatr Child Health (Oxford)* 2011, **16**:March.

28. Sauvaget E, David M, Bresson V, Retornaz K, Bosdure E, Dubus JC: **[Nebulized hypertonic saline and acute viral bronchiolitis in infants: current aspects]. [Review] [French]**. *Arch Pediatr* 2012, **19**:635–641.
