# Supplementary material for: Hypertonic saline (HS) for acute bronchiolitis: Systematic review and meta-analysis
Source: BMC Pulm Med. 2015 Nov 23;15:148. doi: 10.1186/s12890-015-0140-x (PMC4657365; doi:10.1186/s12890-015-0140-x)
Supplement: Additional file 7: — Clinical issues. (DOCX 34 kb) [file 12890_2015_140_MOESM7_ESM.docx]

**Study characteristics**

| **Study/Country** | **Population** | **Intervention** | **Comparator(s)** | **Outcomes (Primary, Secondary)** |
| --- | --- | --- | --- | --- |
| Al-Ansari 2010 et al / Qatar (Single centre) [67] | Inclusion: Children under 18 months, diagnosed with acute bronchiolitis, upper respiratory tract infection, wheezing and/or crackles on auscultation, BSS (> 4).  Exclusion: <34 weeks gestation, history of wheezing, steroid use within 48 hours, obtundation and progressive respiratory failure requiring ICU admission, apnea within 24 hours, oxygen saturation <85% in air, chronic lung disease, congenital heart disease, immunodeficiency. | 3% Hypertonic saline (HS) (5ml), 5% Hypertonic saline (HS) (5ml). Administered every 4 hours until they were ready for discharge, via a nebuliser (flow rate 10L/min). Administered with 1.5ml epinephrine. | 0.9% Normal saline (NS) (5ml) administered every 4 hours until they were ready for discharge via a nebuliser (flow rate 10L/min). Administered with 1.5ml epinephrine. | Bronchiolitis severity score improvement at 48 hours, length of hospital stay, readmissions within 7 days and adverse events. |
| Espelt et al 2012 / Argentina (number of centres unknown)  [25] | Inclusion: Infants aged 1 to 24 months, 1st episode of bronchiolitis with clinical severity score of >5 and 02 saturation of <97%**.**  Exclusion: Chronic respiratory or cardiovascular disease, respiratory failure. | 3% Hypertonic saline (HS) (3ml), administered 4 times per day over 5 days via a nebuliser. Administered with 0.25 mg/kg Albuterol. | 0.9% saline (NS) (3ml), administered 4 times per day over 5 days via a nebuliser. Administered with 0.25 mg/kg Albuterol. | Length of hospital stay and length of oxygen use. |
| Everard et al 2014 / UK (Multi-centre) [72] | Inclusion: Previously healthy infants under 1 year of age, admitted with acute bronchiolitis, oxygen saturation <92% in air, consented and randomised within 4 hours of admission by a medical paediatrician.  Exclusion: Wheezy bronchitis or asthma – children with an apparent viral respiratory infection and wheeze with no or occasional crepitations, reflux, previous lower respiratory tract infections (requiring assessment in hospital), risk factors for severe disease [gestation <32 weeks, immunodeficiency, neurological and cardiac conditions, chronic lung disease], subjects where the carer’s English is not fluent and translational services are not available. | 3% Hypertonic Saline (HS) (4ml), administered every 6 hours until fit for discharge via PARI Sprint nebuliser (flow rate 5L/minute). | Usual Care | Time to fit for discharge, time to actual discharge (length of hospital stay), admission to intensive care units, readmission rates, duration of respiratory symptoms within 28 days of randomisation, healthcare utilisation within 28 days of randomisation, Infant and parental quality of life using the Infant Toddler Quality of Life (ITQoL) questionnaire at 28 days following randomisation and adverse events. |
| Giudice et al 2012 / Italy (Single centre) [61] | Inclusion: Children under 2 years, first episode of bronchiolitis, 02 saturation of <94% in room air, significant respiratory distress (Wang CSS score).  Exclusion: Pre-existing cardiac/pulmonary disease, premature birth (<36 wks), previous asthma diagnosis, initial 02 stats less than 85% or respiratory distress requiring resuscitation. | 3% Hypertonic saline (HS) administered every 6 hours via a nebuliser (flow rate 6L/min). Administered with aerosolised epinephrine (1.5mg). | 0.9% saline (NS), administered every 6 hours via a nebuliser (flow rate 6L/min). Administered with aerosolised epinephrine (1.5mg). | Length of hospital stay and clinical response determined using clinical severity scores. |
| Kuzik et al 2007 / Abu Dhabi and Canada (Multi centre) [20] | Inclusion: children under 18 months, first episode of bronchiolitis.  Exclusion: Previous history of wheezing, cardiopulmonary disease or immunodeficiency, critical illness requiring admission to ITU, use of nebulised HS in last 12 hrs or premature birth (<34wks). | 3% Hypertonic saline (HS) (4ml) administered every 2 hours (3 doses), every 4 hours (5 doses), every 6 hours till discharge via a nebuliser. | 0.9% saline (NS) (4ml), administered every 2 hours (3 doses), every 4 hours (5 doses), every 6 hours till discharge via a nebuliser. | Length of hospital stay and adverse events. |
| Luo et al 2010 / China (Single centre) [62] | Inclusion: children under 2 years, first episode of viral bronchiolitis, mild to moderate bronchiolitis.  Exclusion: >24 months, previous wheezing episode, chronic cardiac or pulmonary disease, immunodeficiency, accompanying respiratory failure, requiring mechanical ventilation, having intervention 12 h before treatment, premature infants. | 3% hypertonic saline (4ml), 3 Treatments every day every 8 hours until discharge via a nebuliser. Administered with 2.5 mg salbutamol. | 0.9% saline (NS) (4ml), 3 Treatments every day every 8 hours until discharge via a nebuliser. Administered with 2.5 mg salbutamol. | Length of hospital stay, cough and wheezing, pulmonary moist crackles remission time and adverse events. |
| Luo et al 2011 / China (Single centre) [63] | Inclusion: infants aged <24 months, first episode of wheezing, admitted to hospital for treatment of moderate to severe bronchiolitis.  Exclusion: >24 months, previous episode of wheezing, chronic cardiac and pulmonary disease, immunodeficiency, accompanying respiratory failure needing mechanical ventilation, inhaled 3% HS 12 hours before treatment, premature birth. | 3% hypertonic saline (HS) (4ml), administered every 2 hours (3 doses), every 4 hours (5 doses), every 6 hours till discharge via a nebuliser. | 0.9% saline (NS) (4ml), administered every 2 hours (3 doses), every 4 hours (5 doses), every 6 hours till discharge via a nebuliser. | Length of hospital stay, clinical severity scores and remission of cough, wheezing, pulmonary moist crackles and adverse events. |
| Maheshkumar et al 2013 / India (Single centre) [66] | Inclusion: Children under 2 years, 1st episode of bronchiolitis, moderate distress.  Exclusion: pre-existing cardiac disease, previous wheezing episode, severe disease (score of >8) needing mechanical ventilation, saturation <85% on room air, cyanosis, obtunded consciousness and/or progressive respiratory failure. | 3% Hypertonic saline (HS) (3ml), administered every 6 hours till fit for discharge, via a nebuliser (flow rate 5-6L/min). Administered with 0.15 mg/kg Salbutamol. | Normal saline (NS) (3ml), administered every 6 hours till fit for discharge via a nebuliser (flow rate 5-6L/min). Administered with 0.15 mg/kg Salbutamol. | Length of hospital stay, reduction in clinical severity scores, number of add on nebulisations and number of treatment failures. |
| Mandelberg et al 2003 / Israel (Single centre) [18] | Inclusion: children up to 12 months, viral bronchiolitis with temperature of >38 degrees C leading to hospitalisation.  Exclusion: cardiac or chronic respiratory disease, previous wheezing episode, saturation <85% in room air, obtunded consciousness, progressive respiratory failure needing mechanical ventilation. | 3% hypertonic saline (HS) (4ml), administered 3 times a day every 8 hours until ready for discharge via an aeromist nebuliser (flow rate 5L/min).Administered with 1.5 mg epinephrine. Administered until nebuliser empty. | 0.9% saline (NS) (4ml), administered 3 times a day every 8 hours until ready for discharge via an aeromist nebuliser (flow rate 5L/min). Administered with 1.5 mg epinephrine. The nebuliser was administered until empty. | Length of hospital stay, change in clinical severity scores and adverse events. |
| Nemsadze et al 2013 / Georgia (Number of centres unknown) [68] | Children 2 months to 2 years old with bronchiolitis, eligibility criteria unclear as abstract only. | 3% Hypertonic saline (HS), administered every 6 hours. | Normal saline (NS) administered every 6 hours. | Bronchiolitis Clinical Score, length of hospital stay and change in oxygen saturation. |
| Ojha et al 2014 / Nepal (Single centre) [71] | Inclusion: Aged 6 weeks – 24 months, presenting with bronchiolitis for the first time.  Exclusion: Previous episode of wheezing, chronic cardiac and pulmonary disease, immunodeficiency, respiratory failure, mechanical ventilation, inhaling nebulised 3% saline and salbultamol 12 hours before treatment, premature infants (less than 34 weeks), oxygen saturation below 85% on room air. | 3% Hypertonic Saline (HS) (4ml), patients in each group received minimum of three nebulization each day delivered at 8 hour intervals until discharge via a nebuliser. | 0.9% Normal Saline (NS) (4ml), patients in each group received minimum of three nebulisation each day delivered at 8 hour intervals until discharge via a nebuliser. | Length of hospital stay and requirement of oxygen supplementation. |
| Ozdogan et al 2014 / Turkey (Number of centres unknown) [27] | Inclusion: Infants 1-24 months of age admitted to hospital with acute bronchiolitis.  Exclusion: unclear – abstract only. | 3% Hypertonic Saline (HS), 5% Hypertonic Saline (HS). Nebulisations given three times a day via a nebuliser, albuterol was given with each dose. | 0.9% normal saline (NS) administered via nebulisations given three times a day, each dose was administered with Albuterol. | Change in respiratory score and length of hospital stay. |
| Pandit et al 2013 / India (Single centre) [65] | Inclusion: 2 to 12 months and admitted with acute bronchiolitis.  Exclusion: Previous wheezing and respiratory distress, family history of asthma, atopy, congenital heart disease, ventilation as new-born, patients with shock, seizures, heart rate (>180/min), respiratory rate (>100/min) and in respiratory failure, consolidation lung on X-ray. | 3% Hypertonic saline (HS) (4ml). The nebulisation was given three times with an interval of one hour, there after nebulisation was given every 6 hours until discharge via a nebuliser (flow rate 6-8 L/minute). Administered with 1ml/1mg Adrenaline. | 0.9% saline (NS) (4ml). The nebulisation was given three times with an interval of one hour, there after nebulisation was given every 6 hours until discharge via a nebuliser. Administered with 1ml/1mg Adrenaline. | Length of hospital stay, improvement in Respiratory Distress Assessment Instrument score, haemoglobin saturation, respiratory rate, heart rate, number of add on treatments and adverse events. |
| Sharma et al 2013/ India (Number of centres unknown)[64] | Inclusion: Children from 1- 24 months,1st episode of acute bronchiolitis, hospitalised, clinical severity score 3-6. Exclusions: obtunded consciousness, cardiac disease, chronic respiratory disease, previous wheezing episode, progressive respiratory distress needing respiratory support other than oxygen. | 3% Hypertonic saline (HS) (4ml), administered every 4 hours, 6 times a day till ready for discharge via a nebuliser (flow rate 7 L/minute). Administered with 2.5 mg salbutamol. Nebuliser was administered until empty. | 0.9% saline (NS) (4ml), administered every 4 hours, 6 times a day till ready for discharge via a nebuliser (flow rate 7 L/minute). Administered with 2.5 mg salbutamol. The nebuliser was administered until empty. | Length of hospital stay, clinical severity scores and adverse events. |
| Silver et al 2014 / New York (Number of centres unknown) [70] | Inclusion: Patients up to 12 months of age, admitted to hospital with bronchiolitis.  Exclusion: Status asthmaticus, chronic cardiopulmonary disease, Trisomy 21and immunodeficiency or transplant recipient or neuromuscular disease. Admission directly to ICU, previous use of nebulized hypertonic saline less than 12 hours prior to presentation and previous enrolment in the study in 72 hours prior to presentation. | 3% Hypertonic Saline (HS) (4ml), administered every 4 hours until discharge. Administered using a standard pressurised hospital wall nebuliser flow rate 5L/min and administered until nebuliser treatment complete. | 0.9% normal saline (NS) (4ml), administered every 4 hours until discharge via a nebulizer with 5 litres oxygen flow. Administered until nebuliser treatment complete. | Length of hospital stay, readmission for bronchiolitis within 30 days of discharge and adverse events. |
| Sosa-Bustamante et al 2014 / Mexico (Single centre) [26] | Inclusion: Aged 2-24 months, first episode of wheezing associated with respiratory distress, history of upper respiratory tract infections and evaluation of respiratory difficulty with Scale Hospital Sant Joan de Deu from 6 – 16 at entry points.  Exclusion: Subjects with history of previous wheezing, asthma, or who have received bronchodilator treatment before the present illness. Patients with chronic lung disease, heart disease, with congenital or acquired anatomic abnormalities of the airway. | 3% Hypertonic Saline (HS) (4ml), nebulized for 3 initial sessions lasting 20 minutes each and every 4 hours during hospital stay, 100 micrograms / kg salbutamol was given with each dose. | 0.9% Hypertonic Saline (NS) (4ml), administered via nebulisations for 3 initial sessions lasting 20 minutes each and every 4 hours during hospital stay. Administered with 100 micrograms / kg salbutamol. | Score respiratory distress, hours of hospital stay, hospital readmission and frequency of complications of the disease itself. |
| Tal et al 2006 / Israel (Number of centres unknown) [19] | Inclusion: children under 12 months, clinical presentation of viral bronchiolitis that led to hospitalization.  Exclusion: Cardiac disease, chronic respiratory disease, previous wheezing episode, age >12 months, 02 sats <85%, obtunded consciousness and/or progressive respiratory failure needing ventilation. | 3% Hypertonic saline (HS) (4ml), 3 treatments each day administered every 8 hours via a nebuliser (output 0.25 ml/min). Administered with 1.5mg epinephrine. Nebuliser administered until empty. | 0.9% Saline (NS) (4ml), 3 treatments each day administered every 8 hours via a nebuliser (output 0.25 ml/min). Administered with 1.5mg epinephrine. The nebuliser was administered until empty. | Length of hospital stay, change in clinical severity scores and adverse events. |
| Teunissen et al 2014 / The Netherlands (Multi-centre) [69] | Inclusion: Children aged 0-24 months, admitted to hospital with viral bronchiolitis with a Wang score of >3.  Exclusion: Excluded if Wang score improved by at least 2 points after inhalation, congenital heart disease, chronic pre-existent lung disease, T-cell immunodeficiency, corticosteroid treatment and previous wheezing, eczema or food allergy. | 3% Hypertonic saline (HS) (4ml), 6% Hypertonic saline (HS) (4ml). Administered every 8 hours until discharge via a HOT Top Plus Nebuliser (flow rate 6-8 L/minute). Administered with 2.5mg salbutamol. Nebuliser administered until empty. | 0.9% saline (NS) (4ml), administered every 8 hours until discharge via a HOT Top Plus Nebuliser (flow rate 6-8 L/minute). Administered with 2.5 mg salbutamol. The nebuliser was administered until empty. | Length of hospital stay, clinical severity scores, transfer to Paediatric Intensive Care Units, need/duration of tube feeding or supplemental oxygen and adverse events. |
