# Supplementary material for: Hypertonic saline (HS) for acute bronchiolitis: Systematic review and meta-analysis
Source: BMC Pulm Med. 2015 Nov 23;15:148. doi: 10.1186/s12890-015-0140-x (PMC4657365; doi:10.1186/s12890-015-0140-x)
Supplement: Additional file 8: — Risk of bias. (DOCX 21 kb) [file 12890_2015_140_MOESM8_ESM.docx]

**Clinical Issues**

| **Study** | **Upper age (months)** | **Mean age at baseline**  **(months)** | **Time to randomise/receive intervention from admission (hours)** | **Clinical criteria required for inclusion** | **Clinical score required for inclusion** | **Oxygen saturations required for inclusion (%)** | **Mean severity scores at baseline entry** | **Mean oxygen saturations at baseline (%)** |
| --- | --- | --- | --- | --- | --- | --- | --- | --- |
| Al-Ansari et al 2010 [67] | 18 | 0.9% NS: 3.3  3% HS: 3.8  5% HS: 4.0 | NR | Wheeze and/or crackles | Wang bronchiolitis severity score >4 | NR | 0.9% NS: 5.77  3% HS: 6.16  5% HS: 5.65 | 0.9% NS : 97.4  3% HS: 97.0  5% HS: 97.4 |
| Espelt et al 2012 [25] | 24 | 0.9% NS: 24  3% HS: 24 | NR | NR | Severity score >5 | < 97 | NR | NR |
| Everard et al 2014 [72] | 12 | Standard care: 3.4  3% HS: 3.3 | < 4 | Crackles | NR | <92 | NR | NR |
| Giudice et al 2012 [61] | 24 | 0.9% NS: 4.2  3% HS: 4.8 | <12 | First episode of wheezing with sign of viral infection. | NR | <94 | 0.9% NS: 8.8  3% HS: 8.5 | 0.9%NS: 92.8  3% HS: 93.5 |
| Kuzik et al 2007 [20] | 18 | 0.9% NS: 4.6  3% HS: 4.4 | <12 | Wheeze and or crackles | RADI >4 | <94 | 0.9% NS: 8.1  3% HS: 7.8 | 0.9% NS: 95.2  3% HS: 94.9 |
| Luo et al 2010 [62] | 24 | 0.9% NS: 5.6  3% HS: 6.0 | NR | First episode of wheezing | Wang clinical score < 8.9 | NR | 0.9% NS: 5.7  3% HS: 5.8 | NR |
| Luo et al 2011 [63] | 24 | 0.9% NS: 5.8  3% HS: 5.9 | NR | First episode of wheezing | Wang clinical score >5 | NR | 0.9% NS: 8.5  3% HS: 8.8 | NR |
| Maheshkumar et al 2013 [66] | 24 | Overall: 5.93 | NR | Bronchiolitis | Wang clinical score between 4 and 8 | NR | 0.9% NS: 6.05  3% HS: 5.55 | NR |
| Mandelberg et al 2003 [18] | 12 | 0.9% NS: 2.6  3% HS: 3 | <24 | Bronchiolitis with temperature >38C | NR | NR | 0.9% NS: 8.08  3% HS: 8.29 | 0.9% NS: 94.7  3% HS: 93.8 |
| Nemsadze et al 2013 [68] | 24 | NR | NR | NR | NR | NR | NR | NR |
| Ojha et al 2014 [71] | 24 | 3% HS: 8.61  0.9% NS: 8.51 | <24 | Presentation of bronchiolitis for the first time | NR | NR | 3% HS: 8.08 Clinical score  0.9%NS: 7.36 Clinical score | 3% HS: 91.47  0.9%NS: 90.58 |
| Ozdogan et al 2014 [27] | 24 | Overall: 7.1 | NR | NR | NR | NR | NR | NR |
| Pandit et al 2013 [65] | 12 | NR | NR | First episode of wheezing | NR | NR | 0.9% NS: 11.7 RDAI  3% HS: 12 RDAI | 0.9% NS: 90.57  3% HS: 90.82 |
| Sharma et al 2013 [64] | 24 | 0.9% NS: 4.18  3% HS: 4.93 | <24 | First episode of wheezing | Wang clinical score 3 - 6 | NR | Median 0.9% NS: 6  Median 3% HS: 6 | 0.9% NS: 95.23  3% HS: 94.43 |
| Silver et al 2014 [70] | 12 | 3% HS: 3.9  0.9% NS: 4.4 | NR | Bronchiolitis | NR | NR | 0.9% HS: 3.2 RDAI  3% NS: 3.5 RDAI | NR |
| Sosa-Bustamante et al 2014 [26] | 24 | NR | NR | First episode of wheezing | Hospital Sant Joan de Deu (HSJD) from 6 to 16 | NR | NR | NR |
| Tal et al 2006 [19] | 12 | 0.9% NS: 2.3  3% HS: 2.8 | <24 | Bronchiolitis | NR | NR | 0.9% NS: 7.6  3% HS: 7.4 | 0.9% NS: 92.9  3% HS: 93 |
| Teunissen et al 2014 [69] | 24 | 0.9% NS: 3.6  3% HS: 3.6  6% HS: 3.4 | <12 | Wheezing | Wang clinical score >3 | NR | 0.9% NS: 6.2  3% HS: 6.2  6% HS: 6.2 | 0.9% NS: 95.5  3% HS: 95.4  6% HS: 95.4 |
