# Supplementary material for: Hypertonic saline (HS) for acute bronchiolitis: Systematic review and meta-analysis
Source: BMC Pulm Med. 2015 Nov 23;15:148. doi: 10.1186/s12890-015-0140-x (PMC4657365; doi:10.1186/s12890-015-0140-x)
Supplement: Additional file 9: — Adverse events narrative. (DOCX 22 kb) [file 12890_2015_140_MOESM9_ESM.docx]

**Risk of bias**

| **Study** | **Allocation concealment** | **Randomisation** | **Blinding of patients and personnel** | **Blinding of outcome assessment - primary outcome** | **Intention to treat (ITT) and withdrawals** | **Selective outcome reporting** |
| --- | --- | --- | --- | --- | --- | --- |
| Al-Ansari et al 2010 [67] | Low: “computer generated list of random numbers was used by the enrolling physicians in consecutive order to identify a sealed envelope”. | Low: computer generated list | Low: double blind | Unclear: no statement provided | High: 16 patients excluded from analysis, does not state which arm each of the patients were allocated to. 171 out of 187 included (91%). | Unclear: insufficient information to make this judgement |
| Espelt et al 2012 [25] | Unclear: no description given | Unclear: no description given | High : "open label" | Unclear: no statement provided | High: 13 withdrawals from intervention group and 5 from control, no reasons given for uneven distribution. 82 out of 100 randomised included (82%). | Unclear: insufficient information to make this judgement |
| Everard et al 2014 [72] | Low: web based randomisation system | Low: computer generated | High: non-blinded | Unclear: Doctor making decision for discharge may not be aware of the treatment allocation | Low: 26 excluded from analysis as randomised when ineligible, 16 of which from the HS group and 10 from the control. All treatment withdrawals from HS group, however the other arm was usual care. 3 withdrawals from the study, 2 of which from control and 1 from HS group. 291 out of 317 included (92%). | Low: All outcomes reported as stated in the protocol. |
| Giudice et al 2012 [61] | Low: study solutions prepared by local pharmacy | Low: computer sequence | Low: double blind | Unclear: no statement provided | Low: 3 withdrawals in total across both groups. 106 of 109 patients followed up (97%). | Low: outcomes reported same as those listed on clinical trials.gov |
| Kuzik et al 2007 [20] | Low: identical appearance drug containers in sequence, research pharmacist prepared solutions | Low: computer sequence | Low: double blind | Unclear: no statement provided | Low: 5 withdrawn (2 intervention and 3 control group). 100% included in ITT analysis. | Unclear: insufficient information to make this judgement |
| Luo et al 2010 [62] | Unclear: "patients were selected and randomly assigned" | Unclear: no description given | Low: double blind | Low: Attending physicians who made decision to discharge blinded to intervention | Low: all patients included in analysis. 100% included in final analysis. | Unclear: insufficient information to make this judgement |
| Luo et al 2011 [63] | Low: opaque, sealed envelope | Low: computer sequence | Low: double blind | Low: Attending physicians who made decision to discharge blinded to intervention | Low: 14 patients excluded as discharged within 12 hours of enrolment. 112 of 126 patients included in final analysis (89%). | Unclear: insufficient information to make this judgement |
| Maheshkumar et al 2013 [66] | Unclear: "patients were recruited sequentially and randomized" | Low: computer sequence | Low: double blind | Unclear: no statement provided | Low: no patients withdrawn, 100% included in the analysis. | Unclear: insufficient information to make this judgement |
| Mandelberg et al 2003 [18] | Unclear: "patients were selected and randomly assigned" | Unclear: no description given although it mentions "the code was deposited with the statistician" | Low: double blind | Low: Attending physicians who made decision to discharge blinded to intervention | Low: 9 withdrawals (3 from intervention and 6 from control; 8 due to withdrawal of parental consent and 1 due to clinical deterioration). 52 of 61 randomised included (85%). | Unclear: insufficient information to make this judgement |
| Nemsadze et al 2013 [68] | Unclear: “Infants were randomly assigned” | Unclear: No description given - abstract only | Unclear: No statement provided -abstract only | Unclear: No statement provided - abstract only | Unclear: No description given - abstract only. | Unclear: insufficient information to make this judgement – abstract only |
| Ojha et al 2014 [71] | Low: Allocation kept in sealed envelopes relating to identical containers and labelled as solution A and B | Low: computer generated random number table, | Low: double blind | Unclear: no description given | Low: 5 excluded from NS group and 8 excluded from HS group, all accounted for. 59 out of 72 included (82%). | Unclear: insufficient information to make this judgement |
| Ozdogan et al 2014 [27] | Unclear: No description given - abstract only | Unclear: No description given - abstract only | Low: double blind | Unclear: No description given - abstract only | Unclear: No description given - abstract only | Unclear: insufficient information to make this judgement |
| Pandit et al 2013 [65] | Low: “Group allocation concealed in opaque envelope” | Low: computer sequence | High: “non-blinded study” | Unclear: no statement provided | Low: no patients withdrawn, all included in analysis. “Out of 100 cases there were no drop outs” | Unclear: insufficient information to make this judgement |
| Sharma et al 2013 [64] | Low: identical appearance containers | Low: computer sequence | Low: double blind | Unclear: no statement provided | Low: 2 withdrawals accounted for. 248 of 250 randomised included (99%). | High: Final severity scores (secondary analysis) not fully reported only stated "did not show statistically significant differences in [both] groups" |
| Silver et al 2014 [70] | Low: Web based randomisation system | Low: computer generated in blocks of 8 | Low: double blind | Low: Outcome assessor blinded | Low: 20 “withdrawn” in HS group (10 for clinical worsening, 3 albuterol given, 3 parental requests and 3 protocol deviation. 17 “withdrawn” in NS group (9 for clinical worsening, 5 albuterol given and 3 protocol deviations. 190 out of 227 included (84%). | Unclear: insufficient information to make this judgement |
| Sosa-Bustamante et al 2014 [26] | Unclear: no description given | Unclear: no description given | Low: double blind | Unclear: no description given | Unclear: no results given | Unclear: insufficient information to make this judgement |
| Tal et al 2006 [19] | Low: identical appearance drug containers in sequence | Low: computer sequence, "code deposited with statistician" | Low: double blind | Low: Attending physicians who made decision to discharge blinded to intervention | Low: 3 patients withdrawn. 41 of 44 patients included in analysis (93%). | Unclear: insufficient information to make this judgement |
| Teunissen et al 2014 [69] | Low: identical appearance containers | Unclear:  “Randomisation was done per centre and clustered in blocks of six” | Low: double blind | Low: “All participants, care givers and medical staff were blinded.” | Low: 2 withdrawn and 43 excluded from per protocol analysis, all accounted for. 247 out of 292 analysed (85%). | Low: outcomes reported same as those listed on The Dutch Trial Register |
