# Supplementary material for: Hypertonic saline (HS) for acute bronchiolitis: Systematic review and meta-analysis
Source: BMC Pulm Med. 2015 Nov 23;15:148. doi: 10.1186/s12890-015-0140-x (PMC4657365; doi:10.1186/s12890-015-0140-x)
Supplement: Additional file 10: — Discharge criteria. (DOCX 18 kb) [file 12890_2015_140_MOESM10_ESM.docx]

**Adverse events narrative**

There was no mention of adverse events in seven studies [25-27, 61, 66, 68, 71] however Maheshkumar et al stated "hypertonic saline is safe" implying no adverse events were observed and Nemsadze et al was only published as an abstract [68]. Tal et al state that there were no adverse events observed in either of the groups [19]. Kuzik et al described that there were no withdrawals due to adverse events by medical staff however two patients in the intervention group were withdrawn on parental request; one because infant cried vigorously during two inhalations; one because of agitation on one inhalation [20]. Luo et al stated that all patients completed treatment, wheezing and coughing did not worsen over course of treatment [62]. In the second season Luo et al highlight that no infants were withdrawn due to adverse events, coughing and wheezing never worsened during treatment. Five infants (two in intervention group) experienced hoarse voices but this improved after 3-4 days [63]. Mandelberg et al describe that no adverse effects were observed [18]. Pulse rate and room air saturation did not differ between both groups on any day. One patient was excluded from analysis due to need for mechanical ventilation [18]. Sharma et al also concur that no adverse events related to any of the patients were reported by parents, caregivers or treating medical attendants in both groups [64]. Al-ansari et al also state that no safety concerns or adverse reactions were identified [67]. Pandit et al describe adverse events of vomiting and diarrhoea that occurred in 4 infants all of which occurred in the control group, in addition they noted no tremors or paleness during treatment [65]. Teunissen et al reported 95 adverse events in the 3% hypertonic saline group, 119 in the 6% hypertonic saline group and 76 in the normal saline group [69]. Silver et al only stated 9 participants in the 3% hypertonic saline group and 4 in the normal saline group did not complete the study due to adverse event [70]. Additional information provided by the author stated 10 participants in the hypertonic saline arm and 9 in the control arm were transferred to ICU/clinical worsening [70]. Finally for the SABRE trial [72], overall there were 51 adverse events recorded in the hypertonic saline group and 43 in the control group. 6 of which were possibly related to the intervention in the hypertonic saline group. These included one serious adverse event (SAE), bradycardia and desaturation during delivery of the hypertonic saline. The five non-serious adverse events were: bradycardia, desaturation, coughing fit and increased respiratory rate (all of which were resolved within one day); and a chest infection which resolved after 6 days.
